# Amyloid‐related imaging abnormalities (ARIA) in anti‐amyloid therapies for Alzheimer's disease: An update from the Alzheimer's Association ARIA workgroup

**DOI:** 10.1002/alz.71361

**Published:** 2026-04-21

**Authors:** Ellis S. van Etten, Simin Mahinrad, Joshua D. Grill, Stephen Salloway, Alireza Atri, Petrice M. Cogswell, Tammie L. S. Benzinger, Takeshi Iwatsubo, Costantino Iadecola, Cynthia A. Lemere, James A. R. Nicoll, Steven M. Greenberg, Maria C. Carrillo, Clifford R. Jack, Reisa A. Sperling

**Affiliations:** ^1^ Department of Neurology Leiden University Medical Center Leiden the Netherlands; ^2^ Alzheimer's Association Chicago Illinois USA; ^3^ Institute for Memory Impairments and Neurological Disorders University of California Irvine Irvine California USA; ^4^ Warren Alpert Medical School Brown University Providence Rhode Island USA; ^5^ Banner Sun Health Research Institute Sun City Arizona USA; ^6^ Banner Alzheimer's Institute Phoenix Arizona USA; ^7^ Brigham and Women's Hospital Harvard Medical School Boston Massachusetts USA; ^8^ Department of Radiology Mayo Clinic Rochester Minnesota USA; ^9^ Mallinckrodt Institute of Radiology Washington University School of Medicine St. Louis Missouri USA; ^10^ National Center of Neurology and Psychiatry Tokyo Japan; ^11^ Feil Family Brain and Mind Research Institute Weill Cornell Medicine New York New York USA; ^12^ Department of Neurology Brigham and Women's Hospital Harvard Medical School Boston Massachusetts USA; ^13^ Clinical Neurosciences Clinical and Experimental Sciences University of Southampton Southampton UK; ^14^ Department of Neurology Massachusetts General Hospital Harvard Medical School Boston Massachusetts USA

**Keywords:** Alzheimer's disease, amyloid‐related imaging abnormalities, amyloid‐targeting therapies, anti‐amyloid therapies, ARIA, cerebral amyloid angiopathy

## Abstract

In 2011, a workgroup of the Alzheimer's Association Research Roundtable introduced recommendations for detecting and monitoring amyloid‐related imaging abnormalities (ARIA) in Alzheimer's disease (AD) clinical trials. Since then, anti‐amyloid immunotherapies have received regulatory approval for AD treatment and are beginning to enter clinical practice, underscoring the importance of informing healthcare providers, researchers, and patients about ARIA's implications in real‐world settings. In response, the Alzheimer's Association convened a new workgroup to review current knowledge of ARIA, including underlying mechanisms, clinical presentations, associated risk factors, mitigation strategies, radiologic detection methods, patients’ perspectives in treatment decision‐making, and outstanding challenges. Here, we outline key insights from this workgroup, highlighting that effective ARIA detection and monitoring in clinical practice requires adherence to robust protocols to mitigate risks and enhance patient safety. Limited availability of clinical and pathologic data on predictors of symptomatic and severe ARIA underscores the importance of continued real‐world data collection.

## Highlights

- The 2011 Alzheimer's Association Research Roundtable workgroup introduced the term amyloid‐related imaging abnormalities (ARIA) and provided initial guidance on detecting and monitoring ARIA – the main adverse events associated with amyloid‐targeting therapies (ATT).
- With ATT now approved by regulatory bodies and entering clinical practice, the 2024 Alzheimer's Association ARIA workgroup reviewed current evidence, identified gaps, and developed updated recommendations to guide real‐world implementation and future research.
- Recommendations span considerations for ARIA terminology use, mitigation of serious adverse events, treatment indications, and antithrombotic and stroke treatment considerations in the context of ATT.
- Future research should prioritize advancing understanding of ARIA mechanisms, improving detection (such as the development of techniques for earlier and more accurate detection of mild radiographic or evolving ARIA), optimizing management strategies with a focus on preventing serious and symptomatic ARIA, evaluating long‐term clinical outcomes, expanding generalizability, and strengthening real‐world data sharing and associated evidence.

## 1 INTRODUCTION

Since the Alzheimer's Association Research Roundtable (AARR) workgroup published recommendations in 2011 for detecting and monitoring amyloid‐related imaging abnormalities (ARIA) in Alzheimer's disease (AD) clinical trials,^1^ multiple Phase 3 randomized controlled trials (Ph3 RCTs) have investigated the effects of amyloid‐targeting therapies (ATTs) on AD clinical, biomarker, and safety endpoints.^2^, ^3^, ^4^ Two monoclonal anti‐amyloid beta (Aβ) antibodies (lecanemab and donanemab) have been approved by the US Food and Drug Administration (FDA) for the treatment of early‐symptomatic AD^5^, ^6^ and are reimbursed by the US Centers for Medicare & Medicaid Services (CMS), conditional on reporting to an approved registry. Aducanumab, another ATT,^2^ was granted accelerated FDA approval, but its production has since been discontinued.

As of late 2025, lecanemab has been approved in 51 countries and regions,^7^ while donanemab is marketed in 13 countries.^8^ In the United States, Japan, China, and many other countries, donanemab and lecanemab are approved regardless of patients’ apolipoprotein E (APOE) ε4 allele carrier status. However, the European Medicines Agency (EMA) has recommended marketing authorization for lecanemab and donanemab only in individuals with one or no copy of the APOE ε4 allele, given their lower risk of ARIA compared with APOE ε4 homozygotes.^9^, ^10^ Similarly, the United Kingdom (UK) Medicines and Healthcare products Regulatory Agency (MHRA) has approved both agents for patients in the early stages of AD who carry one or no APOE ε4 alleles.^11^, ^12^ In Australia, the Therapeutic Goods Administration (TGA) approved donanemab in May 2025 and lecanemab in September 2025 in adults who are APOE ε4 non‐carriers or heterozygotes.^13^, ^14^ Health Canada also recently approved lecanemab in APOE ε4 non‐carriers or heterozygotes,^15^ although, as in Europe, the UK, and Australia, it is not yet covered by national or government insurance schemes. The US Veterans Affairs hospital system refused to approve treatment for APOE ε4 homozygotes altogether.^16^ These differing regulatory outcomes highlight the central importance of ARIA as both a safety concern and a key determinant of how ATTs are evaluated, authorized, and implemented worldwide.

ARIA is recognized as the primary adverse event associated with ATTs, manifesting as vasogenic edema (ARIA‐E) or as hemorrhagic lesions (ARIA‐H). ARIA was first observed during the bapineuzumab trial, the first passive ATT,^1^, ^17^, ^18^, ^19^, ^20^, ^21^ and has since been observed across other ATT trials. In the Ph3 CLARITY‐AD trial (NCT03887455), ARIA‐E and ARIA‐H occurred in 12.6% and 17.3% of participants on lecanemab, respectively.^4^ In the Ph3 TRAILBLAZER‐ALZ‐2 trial (NCT04437511), ARIA‐E and ARIA‐H occurred in 24% and 31% of participants on donanemab, respectively.^3^ Across these trials, ARIA was most often asymptomatic or mildly symptomatic and detected through routine magnetic resonance imaging (MRI) monitoring.^22^ However, symptomatic ARIA can require hospitalization, specialized management, and prolonged monitoring and in rare cases has resulted in death.^3^, ^23^, ^24^, ^25^, ^26^, ^27^

The 2011 report on ARIA by the AARR workgroup noted that data available at the time were too limited to support strong recommendations; nonetheless, it encouraged continuation of clinical trials with careful monitoring. Key recommendations included MRI protocols for ARIA detection, scanning frequency, reading and reporting standards, and participant exclusion criteria based on the presence of baseline microhemorrhages or active edema.^1^ Since then, advances in ARIA knowledge and recent regulatory approvals of ATTs have highlighted the need to reassess and update these recommendations. In 2024, the Alzheimer's Association convened a new independent workgroup of subject‐matter experts – selected on the basis of their diverse scientific expertise, absence of pharmaceutical industry employment, professional involvement in AD research or cerebral amyloid angiopathy (CAA), and representation across professional settings, geographic regions, and genders – to review the state of the science on ARIA and identify priorities for future research. A full list of members and disclosures is publicly available (alz.org/ARIAworkgroup).

Unlike appropriate use recommendations (AURs) that address specific drugs,^26^, ^27^, ^28^, ^29^ the workgroup aimed to address ARIA with an inclusive framework that spans all ATTs. Its deliberations were drawn based on monthly meetings, structured discussions, and a review of the relevant literature, including peer‐reviewed and publicly available data on clinical trials, clinical practice, natural history observations in AD and aging, and preclinical findings from histopathological and animal models. The workgroup also reviewed existing guidance and recommendations regarding clinical use of FDA‐approved ATTs and ARIA, such as FDA Prescribing Information (FDA‐PI)/labels, AURs, and other relevant published material.

This document summarizes the workgroup's review and deliberations, providing an updated synthesis of the state of the science on ARIA, existing knowledge gaps, and the directions the field should take to address these gaps to advance research and clinical practice. It is intended to inform the field broadly but does not constitute a clinical practice guideline or prescriptive protocol for healthcare providers.

## 2 ARIA DEFINITION

In the 2011 AARR recommendations, the term ARIA was introduced for the first time and broadly defined to encompass both treatment‐emergent and spontaneous events.^1^ The 2024 workgroup proposes refining this definition to refer specifically to MRI signal abnormality that emerges in the context of ATTs. Imaging findings with ARIA‐like features do occur in placebo groups of ATT clinical trials and in untreated populations (see Section 2.1 below for more detail). The workgroup recommends that such ARIA‐like features should not be designated as ARIA; instead, they should be described according to the specific radiologic finding observed (e.g., vasogenic edema/effusion, microhemorrhage, superficial siderosis). The intent of such a distinction is to improve timely and targeted communication of ARIA findings to referring providers, supporting treatment mitigation and optimal patient care.

**FIGURE 1:**
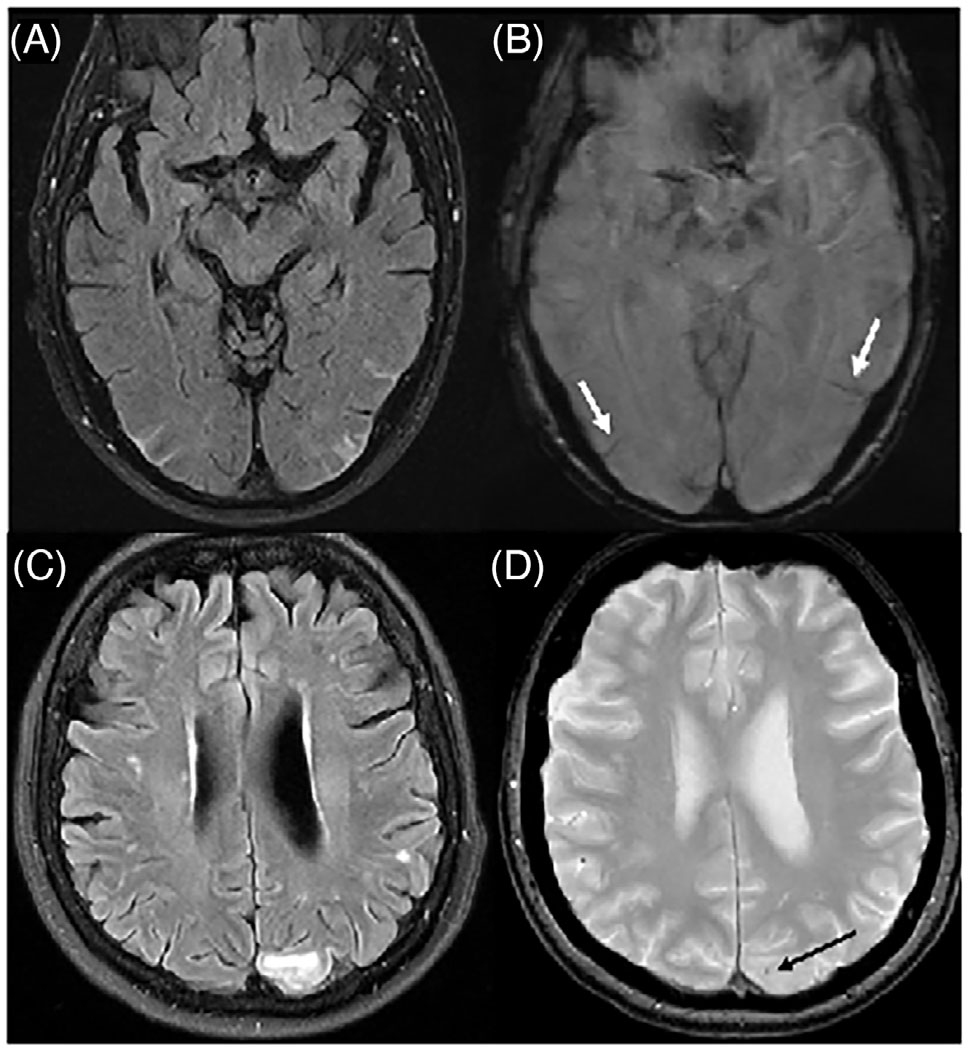
Examples of ARIA‐E and ARIA‐H. Patient 1 (A, B) with moderate ARIA‐E (effusion, edema) and moderate ARIA‐H (siderosis). (A) FLAIR MRI demonstrates hyperintense bilateral occipital and left posterior temporal sulcal effusions and left occipital vasogenic edema. (B) Corresponding susceptibility‐weighted imaging (SWI) has bilateral linear hypointensities, consistent with superficial siderosis (white arrows). The patient had mild symptoms (reported transient blurry vision but denied headache, dizziness, or other symptoms). Dosing was suspended, and the patient was scheduled for a 1‐month follow‐up MRI. Patient 2 (C, D) with mild ARIA‐E and mild ARIA‐H. (C) FLAIR demonstrates hypertense signal and mild mass effect in the superior left occipital measuring up to 2 cm. (D) Gradient echo (GRE) imaging shows one new microhemorrhage in the same region (black arrow). The patient was asymptomatic. Dosing was suspended, and the ARIA‐E resolved on the 1‐month follow‐up MRI. ARIA, amyloid‐related imaging abnormalities; ARIA‐E, ARIA manifesting as vasogenic edema; ARIA‐H, ARIA manifesting as hemorrhagic lesions; FLAIR, fluid‐attenuated inversion recovery; MRI, magnetic resonance imaging.

Accordingly, the workgroup identified the spectrum of treatment‐emergent imaging abnormalities of edema/effusion and hemorrhage to be collectively termed ARIA. Consistent with the 2011 report, two major subtypes were identified to describe specific phenomena underlying ARIA:

- ARIA‐E (Edema): indicative of interstitial vasogenic edema or sulcal effusions in the leptomeningeal/subpial space shown as hyperintensities on T2‐weighted fluid‐attenuated inversion recovery (FLAIR) sequences in patients receiving ATTs (Figure 1).
- ARIA‐H (Hemorrhage): indicative of hemosiderin deposits, including cerebral microhemorrhages and/or superficial siderosis and, rarely, macrohemorrhages on T2*‐weighted imaging (T2*WI) and/or susceptibility‐weighted sequences in patients receiving ATT (Figure 1).

If the treatment status of a patient is unknown (e.g., ongoing participation in a blinded clinical trial) and evidence of edema or effusion is present, then ARIA‐E should be considered and described as per the American Society of Neuroradiology (ASNR) and AUR recommendations^26^, ^27^, ^28^, ^29^, ^30^, ^31^ or with relevant national or regional guidelines applicable to the clinician's practice setting.

The workgroup also considered revising the ARIA terminology to reflect more specific descriptors but ultimately decided to maintain continuity with existing educational material in clinical practice. Additionally, while the workgroup is aware of ARIA‐like phenomena occurring in other AD trials testing non‐amyloid mechanisms, the underlying mechanism of such events is unclear. Therefore, the workgroup determined that, at this time, changes to the terminology would be premature until further evidence becomes available.

### 2.1 Spontaneous hemorrhages and edema

CAA is frequently present in patients with AD. Autopsy studies show that nearly half of individuals diagnosed with AD dementia exhibit moderate to severe CAA using neuropathologic criteria, where its presence contributes to vascular injury and disease progression.^32^ Outside the context of ATTs, spontaneous hemorrhagic and edematous imaging findings can occur as a consequence of underlying CAA. In CAA, Aβ deposition damages vessel wall integrity, leading to hemorrhagic lesions (e.g., microhemorrhages, cortical superficial siderosis [cSS], and intracerebral hemorrhage [ICH]) and non‐hemorrhagic markers (e.g., white matter hyperintensities [WMHs] and enlarged perivascular spaces).^33^ Microhemorrhages associated with CAA typically exhibit the same lobar distribution and appearance as ARIA‐H observed in patients receiving ATTs. In contrast, microhemorrhages related to arteriolosclerosis, which is driven by cardiovascular risk factors, are generally found in the deeper regions of the brain.^34^

Though transient changes on FLAIR imaging suggestive of edema are rare in ATT trials (1.7% to 2.1% in placebo arms), distinguishing ARIA‐E from artifacts remains challenging. Vasogenic edema may occur spontaneously in a subset of patients with CAA who develop CAA‐related inflammation (CAA‐ri). Imaging may reveal cortical microhemorrhages and vasogenic edema or sulcal effusions, features closely resembling ARIA‐H and ARIA‐E. The underlying mechanism of CAA‐ri remains uncertain. Some studies suggest that a spontaneous autoimmune reaction to Aβ generates anti‐Aβ antibodies,^35^ mirroring the antibody‐mediated vascular responses seen with ATTs, whereas others have not found evidence for a specific antibody response.^36^ Histological analyses of CAA‐ri are typically characterized by severe CAA and a predominantly perivascular lymphocytic infiltrate, with features suggesting plaque removal identified in some cases. Given that cortical biopsies and MRI scans are sometimes performed contemporaneously in CAA‐ri, there is a potential opportunity to gain insights into the vascular changes that underlie ARIA‐like changes. Further study of CAA‐ri may therefore offer a valuable model for exploring the pathophysiological mechanisms of ARIA.

Spontaneous microhemorrhages are common in older adults and may reflect underlying CAA or arteriolosclerosis.^37^, ^38^ Current guidelines for ATT eligibility and ARIA‐H severity scoring count all microhemorrhages equally; however, distinguishing their underlying etiology is essential for understanding pathophysiology and refining future recommendations.^31^, ^39^

## 3 PATHOPHYSIOLOGICAL PROCESSES UNDERLYING ARIA

The pathophysiology of ARIA remains incompletely understood due to limitations in biomarkers, imaging tools, human tissue samples, and suitable models.^22^ There have been few *post mortem* studies of patients with AD who were treated with the FDA‐approved anti‐Aβ monoclonal antibodies. The few case studies reported to date seem to reflect the extreme end of the ARIA severity spectrum, which is discussed further in Section 3.1.2 below. However, more extensive neuropathological studies have been performed on patients who received AN1792, the first Aβ immunotherapy trial in humans.^40^ Unlike passive immunotherapy, where anti‐Aβ antibody is provided directly, AN1792 was an active immunotherapy relying on the patient's own immune system to generate anti‐Aβ antibodies using the full‐length Aβ peptide. Despite this difference, both approaches aim to produce plaque‐binding anti‐Aβ antibodies within the central nervous system (CNS), leading to side effects that may be analogous. Indeed, AN1792‐related side effects,^41^ which halted further development of this agent, shared similarities with what was later termed ARIA.^1^ Consequently, consideration of what is known of the pathophysiology of AN1792‐related side effects is likely of relevance to understanding ARIA in the context of the currently FDA‐approved ATTs.

### 3.1 *Post mortem* findings

#### 3.1.1 Insights from active immunotherapy trials: AN1792‐treated patients

In *post mortem* neuropathological studies of patients receiving AN1792, quantitative comparisons with untreated individuals with AD revealed evidence of variable Aβ plaque removal, with some patients showing complete or focal removal of plaques.^42^, ^43^, ^44^ Plaque removal was associated with a relative reduction in tau protein, particularly evidenced by the removal of plaque‐associated dystrophic neurites and straightening of neuronal processes.^43^, ^44^, ^45^, ^46^, ^47^ However, there was also evidence suggesting the continued spread of neurofibrillary tangles through the brain in a Braak‐like distribution, which putatively corresponds with the continued decline in AD patients despite therapy‐mediated plaque removal.^45^, ^48^ Other observations included enhanced clustering of microglia around plaques and evidence of microglial phagocytosis of Aβ plaques.^44^, ^49^ However, where Aβ plaques had been cleared, microglial activation was reduced.^49^

Particularly noteworthy in the context of ARIA are the vascular changes identified in AN1792‐treated patients. Treated participants were observed to have enhanced severity of CAA, potentially reflecting Aβ from disaggregating plaques tracking to the blood vessel walls.^50^ However, this increase in CAA severity seemed transient, with a subsequent reduction observed in patients with longer duration of life after treatment, who also demonstrated greater plaque clearance.^50^, ^51^ This concept of a transient increase in CAA during plaque mobilization is further supported by experimental animal studies.^52^, ^53^, ^54^ Additionally, there was specific evidence for the removal of Aβ from blood vessel walls in the form of increased concentric splitting of arterial walls, interpreted as reflecting the removal of Aβ following damage to the smooth muscle cells of the medium by prior amyloid deposition.^55^ Further vascular changes included an increased number of cortical microhemorrhages, identified by Perls stain for iron, in some AN1792‐treated patients compared with untreated individuals with AD.^50^

Together, these neuropathological findings point to multiple dynamic changes in several features of AD pathophysiology, including Aβ, tau, the vasculature, and microglia, the complete understanding of which is limited by the number of cases and time points available for study.

Neuropathological studies were performed on two patients who developed AN1792 treatment‐related “meningoencephalitis,” which exhibited imaging features that could likely now be termed ARIA.^44^, ^56^ These cases displayed severe CAA within the leptomeninges and cerebral cortex, including in areas of the cortex cleared of plaques, perivascular collections of lymphocytes, cortical microhemorrhages/microvascular lesions (similar to ARIA‐H), and focal white matter signal change on MRI (equivalent to ARIA‐E) corresponding histologically to focal rarefaction of myelinated fibers. Enlarged perivascular spaces were also noted in the white matter – a feature that has been associated histologically and on imaging with severe CAA.^57^ Further studies using MRI scans of *post mortem* brains from individuals receiving AN1792 confirmed that hemorrhagic lesions on imaging corresponded to iron and calcium deposits on histopathology.^51^ Importantly, cortical iron deposition was associated with a lower Aβ plaque area in treated cases, implying that hemorrhage was associated with Aβ removal.^51^

**FIGURE 2:**
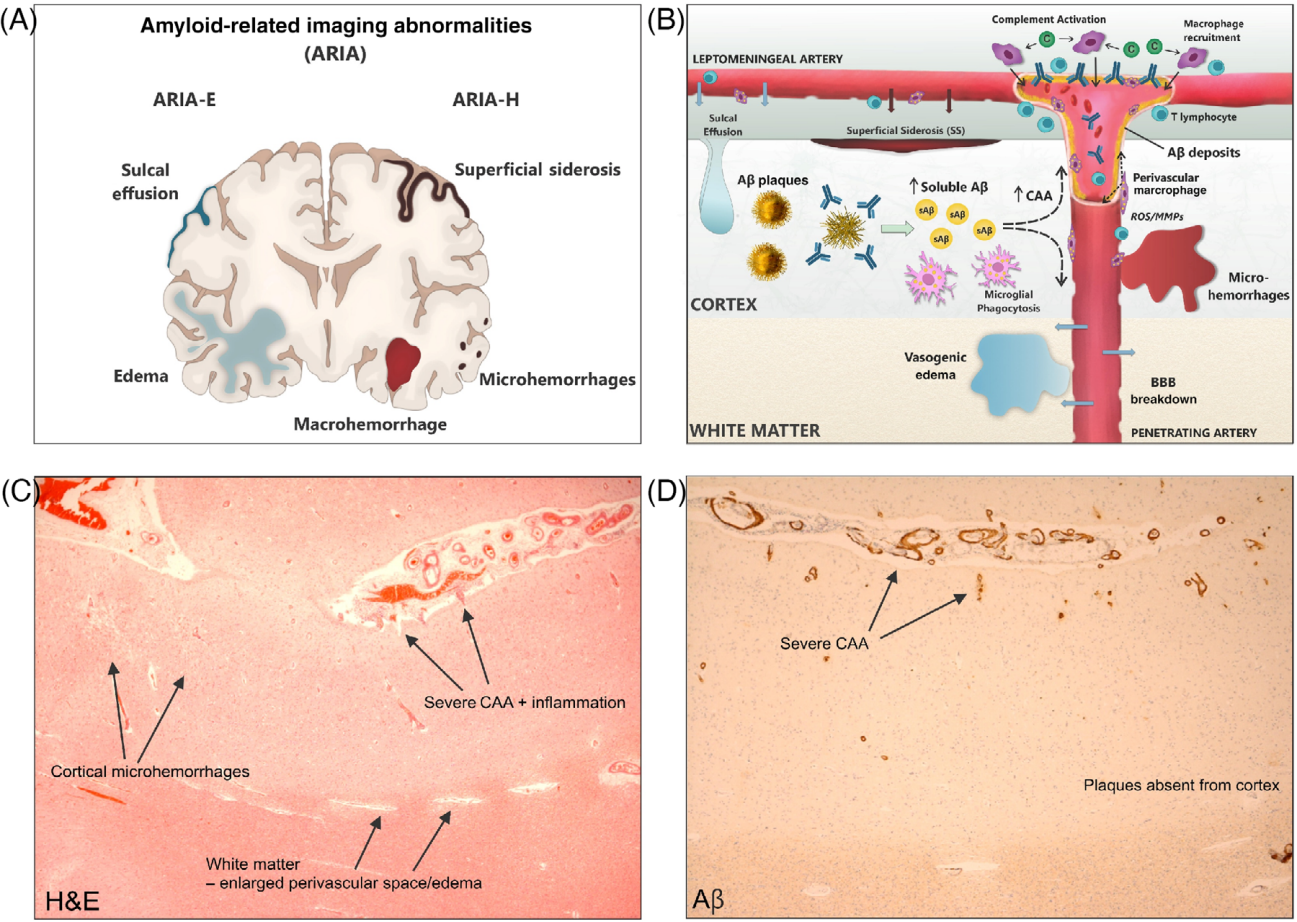
Pathophysiological mechanisms underlying amyloid‐related imaging abnormalities following Aβ immunotherapy. (A) ARIA comprise white matter edema and sulcal effusion (ARIA‐E); cortical microhemorrhages and superficial siderosis and rarely lobar macrohemorrhages (ARIA‐H). (B) Anti‐Aβ antibodies bind plaques and disaggregate amyloid, which is then carried to the vessels for clearance. Antibodies also bind vascular amyloid, activate complement and provoke an inflammatory response involving T‐lymphocytes and macrophages which results in vascular damage. Leakage of antibody entering the brain parenchyma from damaged vessels may enhance plaque removal. ARIA‐H is likely due to damage to the integrity of the walls of small arteries/arterioles in the cortex and leptomeninges affected by CAA. ARIA‐E is likely due to failure to control water flux at the blood–brain barrier as a result of vascular pathology. (C) Anti‐Aβ immunotherapy (AN1792), with arrows indicating histological features corresponding to (b) hematoxylin and eosin. (D) Immunohistochemistry for Aβ showing severe CAA despite the absence of plaques, interpreted as cleared by immunotherapy (artwork by Dr. Jennifer Dewing, University of Southampton, UK). Aβ, amyloid beta; ARIA, amyloid‐related imaging abnormalities; ARIA‐E, ARIA manifesting as vasogenic edema; ARIA‐H, ARIA manifesting as hemorrhagic lesions; CAA, cerebral amyloid angiopathy.

Overall, the information derived from AD patients who received active immunization with AN1792 provided the basis for a proposed pathophysiological mechanism for ARIA as follows^45^ (Figure 2). Antibodies to Aβ enter the cerebral cortex and bind to amyloid plaques, resulting in plaque disruption and tracking of solubilized Aβ to the vasculature, increasing the severity of CAA. Additionally, anti‐Aβ antibodies likely bind directly to amyloid in the walls of vessels within the cerebral cortex and overlying leptomeninges, resulting in the removal of Aβ from the vessel walls affected by CAA. A T‐lymphocyte reaction to vascular changes and the presence of immune complexes may occur. Altogether, these vascular and inflammatory changes can explain the cortical microhemorrhages and superficial siderosis (ARIA‐H). As the affected leptomeningeal arteries form the cortical penetrating arteries, which then proceed to supply the underlying white matter, these vascular changes can result in altered fluid balance in the underlying white matter. The precise mechanism resulting in the white matter edema is not entirely understood but putatively relates to impaired efflux of extracellular fluid by perivascular drainage,^58^ more recently termed intramural periarterial drainage (IPAD).^59^ This overall scheme can, therefore, explain the close proximity of antibody‐mediated removal of Aβ, cortical microhemorrhages (ARIA‐H), and changes in the white matter (ARIA‐E).

#### 3.1.2 Insights from passive anti‐Aβ immunotherapy

The pathophysiology underlying ARIA with passive immunotherapies seems likely to be similar to that observed with AN1792 as described above. A recent study of five participants in a clinical trial of aducanumab included *post mortem* histological evaluation of brain regions that had exhibited ARIA on MRI in two individuals during life.^60^ One person was a 57‐year‐old presenilin‐1 (PS1) mutation carrier who was also heterozygous for APOE ε4. This individual developed moderate ARIA‐E followed by ARIA‐H but continued treatment and came to autopsy 3 years later. The other individual was a 67‐year‐old who was homozygous for APOE ε4 and developed severe ARIA in the open‐label extension (OLE) phase, after having received placebo during the double‐blind phase. This participant experienced clinical decline, discontinued therapy, and came to autopsy 2.5 years later.^60^ ARIA‐affected regions showed distinct pathology with increased CAA, CD8+ T lymphocytes surrounding CAA‐affected vessels, and hemosiderin and microinfarcts primarily located near sulcal leptomeningeal and penetrating vessels. Some blood vessels with CAA showed immunoreactivity for complement C5b‐C9 and CD68, suggesting vessel degradation. No changes were apparent in the white matter. In these aducanumab‐treated patients, evidence for Aβ clearance was most pronounced in the superficial cortex, consistent with access of antibody mainly from the CSF, with relatively little antibody gaining direct access to the brain parenchyma across the blood–brain barrier (BBB).

There is limited direct neuropathological information from ATTs in current clinical use, with *post mortem* studies restricted to individual case reports.^24^, ^25^ As these cases represent unfortunate fatal events, they likely reflect the most severe end of the spectrum of ARIA‐related pathology.

A 65‐year‐old patient (APOE ε4/ε4 genotype) with cognitive decline received three intravenous (IV) infusions of lecanemab^24^ and was evaluated for possible ischemic stroke 4 days after the last infusion. Thrombolysis was initiated, and during the tissue plasminogen activator (t‐PA) infusion, the patient developed multifocal cerebral hemorrhages, with a predominantly cortical distribution, typical of CAA‐related hemorrhage. *Post mortem* analysis revealed hemorrhages were accompanied by CAA with associated histiocytes and lymphocytes and a necrotizing vasculopathy. This patient had no microhemorrhages or superficial siderosis on prior MRI scans. This case illustrates the convergence of multiple risk factors for hemorrhage, including severe CAA, APOE ε4 homozygosity (as a risk factor for both CAA and ARIA), and IV thrombolysis. Patients with severe CAA treated with t‐PA have already been identified as at risk of hemorrhage.^61^, ^62^ However, this case also raises concern over the combination of severe pre‐existing CAA, treatment with Aβ immunotherapy, and thrombolysis.

Another case report of a fatal outcome^25^ involved a 79‐year‐old female (APOE ε4/ε4 genotype) with clinically diagnosed AD who had three IV infusions of lecanemab. After the third infusion, the patient developed a seizure. This patient had moderate to severe WMHs and four microhemorrhages on her screening MRI prior to receiving open‐label lecanemab, and she had headache and confusion following the first two doses of lecanemab. Medications included aspirin and anticoagulation with heparin after identification of atrial fibrillation. Multiple cerebral hemorrhages were identified on MRI. *Post mortem* analysis confirmed the presence of multiple hemorrhages and severe CAA with perivascular lymphocytic infiltrates, reactive macrophages, and fibrinoid degeneration of vessel walls.

A third fatal case with *post mortem* examination has been described in a 75‐year‐old woman (APOE ε4/ε4 genotype) receiving aducanumab in the OLE study.^63^ The patient experienced three episodes of ARIA‐E during a double‐blind trial, while a safety MRI after the seventh dose of OLE aducanumab demonstrated ARIA that was not recognized at the time. She was admitted with focal seizures and rapidly progressed to super‐refractory status epilepticus. MRI showed features consistent with CAA‐ri or ARIA, and biopsy showed severe CAA and inflammation. The patient passed away despite aggressive treatment. Autopsy showed mild to moderate CAA, mild arteriosclerosis without ischemic injury, diffuse reactive gliosis with edema, and numerous activated microglia around amyloid plaques.

Together, these case reports raise important safety concerns about APOE genotype, pre‐existing CAA, and the interaction with concurrent t‐PA and anticoagulant therapy as risk factors for serious ARIA‐H.

### 3.2 Role of immune responses, CAA‐ri, and APOE genotype

The role of the immune response in the generation of ARIA is currently unclear. T lymphocytes have been identified in close relation to CAA‐affected blood vessels in patients developing ARIA after receiving AN1792, aducanumab, and lecanemab. The close analogy between ARIA and the natural disease process of CAA‐ri suggests that these immune cells may have an important role.^35^, ^64^, ^65^, ^66^

The observation that ARIA‐E tends to occur within a few months after initiation of immunotherapy is consistent with the idea that it is closely related to the dynamic phase of plaque removal and accompanying vascular changes. Aβ released from plaques may lead to neurovascular dysfunction and damage by activating scavenger receptors on perivascular and leptomeningeal macrophages, leading to oxidative stress, inflammation, and reduced Aβ clearance.^67^, ^68^

Recent work in animal models^69^ suggests a potential explanation for the co‐localization of ARIA and Aβ removal, which has occasionally been described on imaging of treated patients. In this scheme, vascular inflammation results in increased vessel wall permeability, allowing extravasation of anti‐Aβ antibody from the blood into the brain parenchyma. Consequently, higher levels of parenchymal antibody are available to bind and clear cortical plaques in the vicinity of the inflamed vessels associated with ARIA.

APOE ε4 has been consistently identified as a risk factor for ARIA in clinical studies of immunotherapy. The gene‐dose increase in risk aligns with the proposed mechanism described above, as APOE ε4 is known to be a risk factor for the development of CAA and CAA‐ri.^70^, ^71^ This implies that individuals who have severe CAA as a feature of their AD prior to therapy may be particularly susceptible to these CAA‐related complications. Due to its proposed role as a transport protein for Aβ, APOE may also be involved in the process of antibody‐mediated plaque disaggregation,^55^ subsequent mobilization, and trafficking to the vasculature. Since APOE ε4 leads to neurovascular dysfunction through activation of perivascular and leptomeningeal macrophages,^72^, ^73^ the Aβ reaching the perivascular space may not be efficiently cleared, enhancing vascular inflammation and BBB disruption.

It is worth noting that while most data emphasize APOE ε4 as increasing the risk of ARIA, emerging reports suggest that APOE ε2 may also confer risk for hemorrhagic complications in the setting of ATT. APOE ε2 is protective against AD; however, it has been linked to increased risk of CAA‐related hemorrhages and superficial siderosis.^74^, ^75^ Two cases of ICH have been reported in APOE ε2 carriers receiving lecanemab^76^ and trontinemab,^77^ including a fatal event in an ε2/ε3 individual on trontinemab with baseline MRI features of cSS and a multispot pattern of hyperintensities on FLAIR.^77^ While the link between APOE ε2 and ARIA risk remains to be elucidated, these observations suggest that APOE ε2 status may need to be factored into individual risk assessment of hemorrhagic complications following ATTs.

### 3.3 Relationship of ARIA to ventricular enlargement and brain volume reduction

A consistent finding in clinical trials of ATTs is greater brain volume reduction and ventricular enlargement compared to placebo.^78^ This seems potentially counterintuitive in view of the evidence of treatment‐related slowing of cognitive decline. Explanations include a lowered volume occupied by Aβ plaques, reduced disease‐associated inflammatory changes, fluid shifts, removal of diseased neurons, and treatment‐induced toxicity. The term “amyloid‐removal‐related pseudo‐atrophy” has been proposed for this treatment‐related change to distinguish it from the cerebral atrophy associated with the neurodegeneration.^78^ ARIA‐associated monoclonal antibodies cause accelerated ventricular enlargement compared with anti‐Aβ drugs that do not cause ARIA.^79^ In addition, the increase in ventricular volume is correlated with the percentage of individuals experiencing ARIA with different agents.^79^ A potential confounding factor in this relationship is that ventricular enlargement also correlated with amyloid reduction on PET scans,^79^ so ARIA frequency with an agent may reflect its success in removing plaques. The anatomical localization of ARIA may be associated with focal reductions of amyloid on PET scans,^21^ but the relationship of ARIA with brain volume loss is currently unclear and requires further study.

### 3.4 Non‐clinical models of ARIA

**FIGURE 3:**
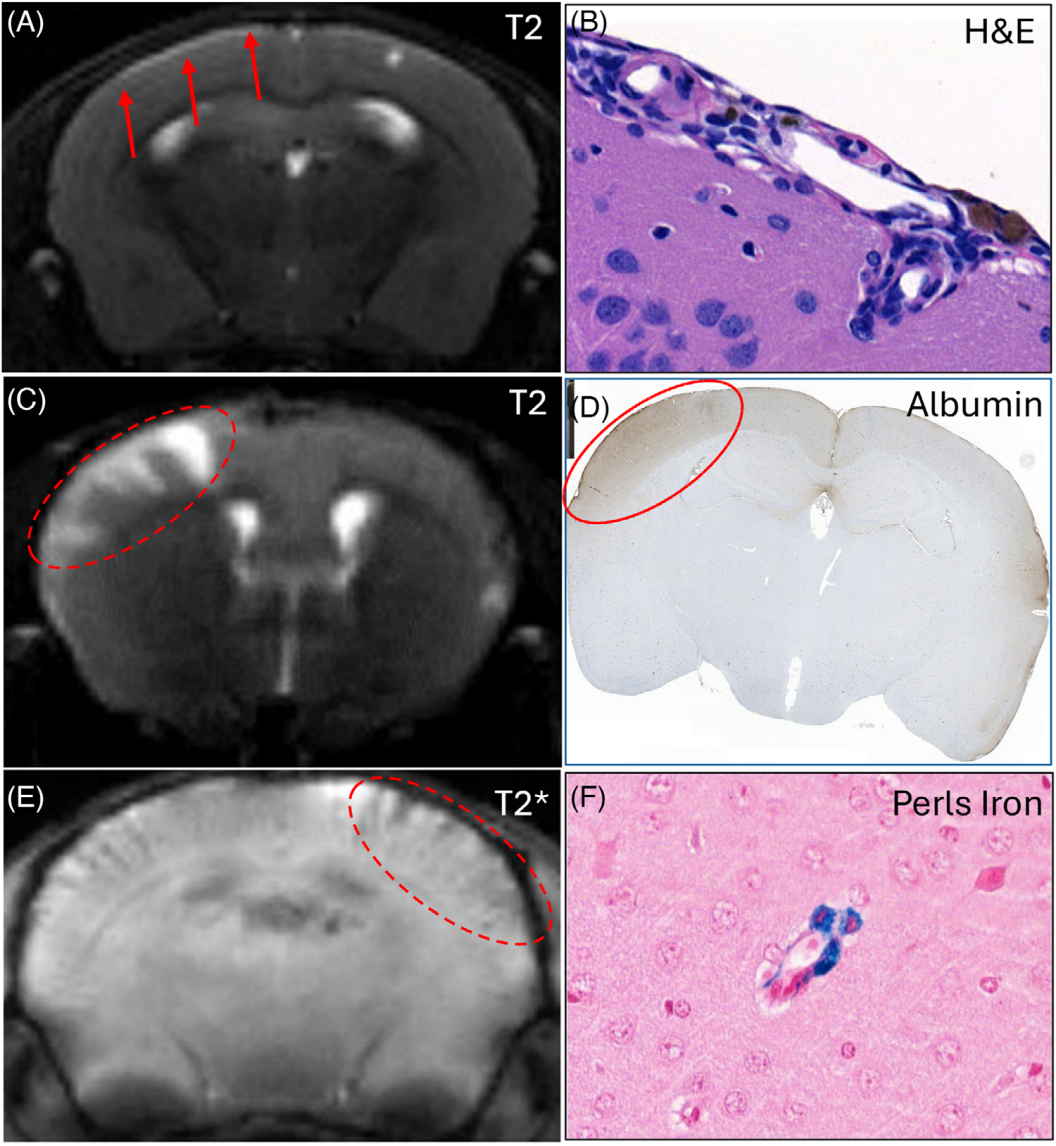
Histological and magnetic resonance imaging features of amyloid‐related imaging abnormalities in mouse models. ARIA‐like lesion observed in 5xFAD transgenic mice treated with 3D6 (5 or 10 mg/kg). ARIA‐E‐like lesions: (a) described as T2 hyperintense convexity lesion (red arrows in A), associated with meningovascular inflammation (B); (b) described as T2 hyperintense focal cortical lesion (red oval in C), associated with albumin leakage within brain parenchyma (red oval in D). ARIA‐H like lesions described as T2* hypointense cortical lesion (red oval in E), associated with Perls iron staining; most likely sequelae of meningovascular inflammation.^86^ Courtesy of Ed Plowey and Thierry Bussiere, Biogen; iCAA Conference, Munich, October 2024, and ADPD/2024, Lisbon, March 2024.

Animal studies dating back to the early 2000s have reported the presence of spontaneous microhemorrhages in CAA‐bearing mouse models, such as the APP23 and Tg2576 transgenic mouse models, which express human amyloid precursor protein (APP) gene mutations. As these mice age, they develop amyloid plaques, followed by strong vascular staining predominantly in meningeal vessels and large penetrating arterioles.^80^ Other models that express human mutant APP and PS1 genes – such as APP_swe_/PS1^dE9^, ARTE10 mice,^82^ APPPS1‐21,^83^ and APP^NL‐F^/Psen1^P117L^ double knock‐in mice^84^ – develop CAA with advanced age. In contrast, 5xFAD mice, which express three human mutant APP genes and two human mutant PS1 genes, develop only modest CAA with aging and no spontaneous microhemorrhages.^85^ Evidence of ARIA‐E and ARIA‐H has been demonstrated in these models by MRI following chronic immunization with 3D6, the murine precursor to bapineuzumab (Figure 3).^86^

The occurrence of anti‐Aβ‐mediated ARIA was predicted by earlier studies in CAA mouse models. Passive Aβ immunization in aged APP23 transgenic mice led to a significant increase in cerebral microhemorrhages, particularly in vessels laden with amyloid.^87^ Similar findings were observed in aged Tg2567 mice, in which passive Aβ immunotherapy improved memory despite increasing CAA and microhemorrhages.^53^

The APP23 and Tg2567 transgenic mouse models develop parenchymal plaques and vessel amyloid primarily composed of Aβ_40_ peptides, leading to the progression of significant levels of CAA and associated microhemorrhages. In contrast, in the PDAPP transgenic mouse model, the parenchymal plaque is primarily composed of Aβ_42_ peptides, similar to human sporadic AD cases. Nonetheless, PDAPP mice also develop age‐dependent CAA, albeit to a lesser extent.^88^ Notably, passive Aβ immunization of aged PDAPP mice with the N‐terminally directed 3D6 antibody, which binds with high affinity to deposited forms of Aβ, exacerbated both the severity and incidence of CAA‐associated microhemorrhages.^88^ In the same mouse model at younger ages, passive immunization with the 3D6 antibody cleared both Aβ plaques and vascular amyloid in a dose‐dependent manner, but also increased the incidence of microhemorrhages, which were limited to focal perivascular sites.^89^

As discussed, the APOE ε4 allele is a significant risk factor associated with both spontaneous CAA and ATT‐related ARIA. Our understanding remains limited by the paucity of human tissue available for study and the difficulty in imaging CAA and inflammation in vivo. However, 5xFAD (low expressor line 7031) mice and APP_swe_/PS1^dE9^ mice on a humanized APOE ε4 background have increased CAA^90^, ^91^ and have been shown to develop numerous microhemorrhages with anti‐Aβ antibody treatment.^92^, ^93^ As for translational implications for human models, emerging human induced pluripotent stem cell‐derived BBB modeling now shows APOE‐genotype effects on Aβ transport and deposition at the BBB, offering a tractable platform to test antibody formats/dosing, Fc engineering, and ARIA‐relevant readouts.^94^

Animal studies have also provided insights into cellular and molecular mechanisms of ARIA. In aged Tg2567 mice with CAA, significant activation of the matrix metalloproteinase (MMP) systems, particularly MMP2 and MMP9, has been observed following treatment with ATTs.^95^ More recently, it was shown that chronic immunization of very old hTau APP KI and PDAPP mice resulted in binding of 3D6 to vascular amyloid, activation of perivascular macrophages, recruitment of peripheral immune cells, upregulation of genes involved in vascular permeability, smooth muscle cell loss, and vascular damage.^96^, ^97^ Fc‐effector activity of anti‐Aβ antibodies can also drive synapse loss and cognitive deficits in AD‐like mice, and strategies that reduce FcγR/C1q engagement are being explored to mitigate ARIA‐like events in preclinical models.^98^, ^99^ Other studies have reported transcriptional changes in subclusters of microglia in APP/PS1 mice following anti‐Aβ immunization with chimeric aducanumab and continued microglial activation in residual plaques; however, after stopping treatment, microglia were not reactivated with the return of cerebral amyloid deposition,^100^ which may have implications for halting treatment in humans after amyloid is cleared. In APP/PS1 mice carrying the human APOE ε4 allele, chronic immunization has also been associated with antibody binding to CAA, activation of the classical complement cascade, local inflammation, and microhemorrhages.^93^

Newer models with earlier and more robust CAA are expected to provide additional insights into ARIA. These include 5XE4 mice (low expressor line 7031) on a human APOE ε4 knock‐in background,^92^ WSB.APP/PS1 mice on a diverse genetic background,^101^ and EC‐APP770+;APP^NL‐F/NL‐F^ mice in which CAA is driven in APP^NL‐F/NL‐F^ mice by the expression of human APP770 in endothelial cells.^102^ Continued investigation in these and related models – particularly in the context of risk factors outlined in Table 1 – may enhance understanding of ARIA pathogenesis and guide treatments.

**TABLE 1:** Established, emerging, and potential risk factors for amyloid‐related imaging abnormalities (ARIA).

ARIA risk factors	Details
**Established ARIA risk factors**	
APOE ε4 status	Higher ARIA risk with more ε4 alleles. Especially elevated risk of ARIA development, severity, recurrence, symptoms, and serious AEs in ε4 homozygotes. ATT FDA‐PI includes a box warning for ARIA that cautions regarding higher and more impactful risks in ε4 homozygotes.
CAA severity – MCHs or cSS on baseline MRI	Higher risk with more MCHs or any cSS on baseline MRI. ARIA risk is greater with one MCH than no MCHs, with two or more MCHs than with one MCH, and with any cSS compared to no cSS.
Treatment timing, dose, and titration	ARIA mostly occurs in the first 2 to 6 months of treatment initiation; risk increases with higher or faster dose escalation. Severe or serious ARIA can, however, occur earlier or later in the treatment period, particularly in persons who are ε4 homozygous.
**Emerging ARIA risk factors**	
High baseline amyloid PET burden and elevated CSF p‐tau	Baseline cortical Aβ‐plaque load of >107 CL on amyloid PET may be associated with higher ARIA‐E risk. In the GRADUATE trial, mean CSF p‐tau concentrations were ∼33 pg/mL in participants who developed ARIA‐E compared with ∼28 pg/mL in those without ARIA‐E.
Higher baseline BP	Baseline MAP > 93 may be associated with marginally higher ARIA‐E risk. Risk of ARIA‐E may be especially higher in persons with MAP ≥ 107.
No use of antihypertensives at baseline, likely reflecting untreated or suboptimally controlled hypertension	Antihypertensive use at baseline may decrease the risk of ARIA‐E.
Baseline WMHs (leukoaraiosis)	Greater WMH burden – as measured by Fazekas score on MRI – may elevate ARIA risk.
**Potential risk factors for severe ARIA and serious complications**	
Anticoagulant use	Anticoagulation could increase the risk of severe ARIA‐H in the form of symptomatic ICH. Safety data regarding concomitant anticoagulation use with ATT remain limited. Several deaths following anticoagulation are reported. FDA labels advise caution regarding anticoagulation, and AURs recommend against concomitant use with ATT until definitive safety date are available.
Administration of thrombolytics	Administration of thrombolytics (e.g., tissue plasminogen activator/tPA) in persons taking ATT carries very high risk. Several deaths following administration of thrombolytics in persons taking ATT are reported. AURs recommend that thrombolysis be avoided in patients on ATT. Box Warning cautions on FDA labels that focus on neurologic symptoms from ARIA‐E may mimic ischemic stroke‐like symptoms and should be considered before potentially giving thrombolytics. AURs advise that mechanical thrombectomy without thrombolytics does not appear to increase ICH risk and should be considered for patients experiencing acute large vessel occlusive stroke.

Abbreviations: Aβ, amyloid beta; AE, adverse event; ARIA, amyloid‐related imaging abnormalities (E: edema, H: hemorrhage); ATT, amyloid targeting therapies; AUR, appropriate use recommendation; BP, blood pressure; CAA, cerebral amyloid angiopathy; CL, Centiloid; CSF, cerebrospinal fluid; cSS, cortical superficial siderosis; FDA‐PI, US Food and Drug Administration Prescribing Information; ICH, intracerebral hemorrhage; MAP, mean arterial pressure; MCH, microhemorrhage; MRI, magnetic resonance imaging; PET, positron emission tomography; p‐tau, phosphorylated tau; WMH, white matter hyperintentsity.

### 3.5 Brainshuttle

In recent years, Brainshuttle technology has emerged as an innovative approach to enhancing the transport of ATTs across the BBB. The platform harnesses transferrin receptor‐mediated transcytosis at the capillary level to deliver therapeutic molecules more efficiently into the brain parenchyma, thereby reducing the dose and enhancing central exposure, target engagement, and biodistribution.^103^ In the context of ARIA, it has been hypothesized that by minimizing interactions with vascular amyloid deposits through a more direct access to the brain via capillaries, this technology may help reduce ARIA risk. Indeed, recent preclinical data demonstrated that a transferrin receptor‐targeted anti‐Aβ antibody engineered using an antibody transport vehicle (ATV) achieved greater parenchymal plaque engagement, enhanced amyloid clearance, and markedly reduced ARIA‐like vascular pathology.^104^

One example of this approach currently in clinical trials is trontinemab. Trontinemab is an anti‐Aβ antibody (gantenerumab) conjugated to a Fab fragment, which binds to a transferrin receptor, facilitating direct transport across the BBB. Initial evidence indicates good BBB penetration and distribution through the brain with effective amyloid clearance. ARIA appears to be infrequent with trontinemab,^103^, ^105^ possibly because of the combination of direct access across capillary walls into the brain parenchyma and relative avoidance of large superficial CAA‐affected blood vessels, which seem mainly responsible for ARIA. Of note, some of the higher‐dose cohorts received a single dose of dexamethasone prior to each treatment to minimize infusion reactions; it remains unknown whether this pre‐treatment has any effect on ARIA occurrence, as lower ARIA rates were also observed among participants who did not receive any steroid pre‐treatment.

These preliminary findings suggest that leveraging Brainshuttle technology represents a compelling strategy in optimizing the therapeutic index of ATTs, with the potential to reduce ARIA risk while maintaining or boosting the efficacy of amyloid clearance.

### 3.6 Subcutaneous formulations of ATT

In addition to IV lecanemeb, subcutaneous (SC) formulations are being developed with the aim of improving patient convenience and safety profiles. Early studies suggested that SC lecanemab may achieve efficacy profiles similar to those of IV administration, while potentially lowering the incidence of ARIA‐E.^106^ Additional studies evaluated a range of SC lecanemab maintenance doses in the Ph3 CLARITY‐AD OLE trial. Results showed that transitioning to SC lecanemab after 18 months of IV therapy maintained clinical and biomarker benefits comparable to continued IV dosing. Rates of ARIA in patients who received SC maintenance dosing were similar to those reported in patients who remained on IV lecanemab after 18 months.^107^ According to these studies, the FDA‐approved SC lecanemab for maintenance dosing following 18 months of IV treatment.^108^ While these studies suggest that SC lecanemab may not increase ARIA risk, continued surveillance and additional studies are needed to further characterize ARIA risk with long‐term SC maintenance dosing.

## 4 RADIOLOGIC MANIFESTATIONS OF ARIA AND DETECTION METHODS

On brain MRI, ARIA‐E is identified on the FLAIR sequence and is characterized by T2 hyperintense signal in the white matter that may involve gray matter. In more extensive cases, there may be associated mass effect, cortical swelling, and effacement of the sulci. ARIA‐E edema is differentiated from acute ischemia by the absence of diffusion restriction. ARIA‐E sulcal effusion is characterized by FLAIR hyperintense signal in the sulci. ARIA‐E is a transient phenomenon, detected only if the timing of the MRI study is correct. ARIA‐E generally resolves within 3 to 4 months as documented by serial monthly MRI scans.^22^ If ARIA‐E does not resolve, further MRI investigation, such as a general brain imaging protocol with and without contrast, should be considered.

ARIA‐H is identified on GRE T2*‐WI and/or susceptibility‐weighted MRI sequences. ARIA‐H often occurs with ARIA‐E in both time and location and may be identified at the same time as ARIA‐E or emerge as ARIA‐E resolves. When ARIA‐H and ARIA‐E co‐occur, ARIA‐H generally occurs as a cluster of regional microhemorrhages with or without siderosis. ARIA‐H may also occur in the absence of a preceding ARIA‐E event, although when this happens, it could simply be that transient ARIA‐E was not captured on a scheduled safety MRI. While ARIA‐E is a transient phenomenon, heme deposits are usually (not always) permanent. Isolated microhemorrhages can also occur in the absence of therapy, likely due to concomitant CAA.^109^, ^110^, ^111^

The FDA recently updated its safety recommendations for lecanemab to include an additional early MRI scan between the second and third infusions, following a safety review that identified two fatal early ARIA‐E cases.^112^ Similarly, the donanemab AUR and its FDA‐PI recommend scheduled safety MRIs before the second, third, fourth, and seventh infusions.^27^, ^113^

In recent trials and per lecanemab and donanemab FDA labels, each ARIA‐E, ARIA‐H microhemorrhage, and ARIA‐H siderosis is graded as mild, moderate, or severe.^113^, ^114^ ARIA severity scoring in trials and clinical practice includes only findings that are new since starting therapy; for example, spontaneous microhemorrhages present on the pre‐treatment baseline exam are not included in the ARIA‐H count. ARIA‐E is graded based on the number of treatment‐emergent contiguous regions of FLAIR signal abnormality and the greatest linear dimension of the largest region. ARIA‐H microhemorrhages and siderosis are graded based on the cumulative number of treatment‐emergent microhemorrhages and regions of superficial siderosis, respectively. Definite, current microhemorrhages and siderosis within an episode of ARIA‐E should be graded, and additionally, cumulative treatment‐emergent (i.e., incident) ARIA‐H needs to be separately tracked. The radiographic ARIA severity score is used along with any patient symptoms to determine management and continued drug dosing.

A standardized and consistent MRI protocol must be used for accurate ARIA detection, monitoring, and severity scoring.^21^, ^30^, ^31^ This includes a standardized set of imaging sequences and sequence parameters performed on similar MRI systems and with similar field strengths. Recent clinical trials have implemented the following set of standardized sequences: 2D FLAIR for ARIA‐E detection, 2D GRE T2* for ARIA‐H detection, 2D DWI to differentiate ARIA‐E and acute ischemia, and 3D T1 for co‐registration between imaging time points and to provide structural information. In trials, these standardized sequences are sent to sites for download, and a specific scanner is approved for use at each site. Therefore, similar imaging is performed across sites and across serial exams. A similar level of standardization and consistency is desired for clinical imaging, and specific recommendations for clinical imaging protocols have been provided by the ASNR Alzheimer's, ARIA, and Dementia Study Group.^30^ Inconsistent parameter selection and scanner models within serial exams for a given patient can result in significant interpretive difficulty, particularly on FLAIR imaging.

One important consideration for clinical practice and future trials is the choice of a hemosiderin‐sensitive sequence. Susceptibility‐weighted sequences are increasingly available and provide improved spatial resolution and higher sensitivity for the detection of blood products compared to gradient‐recalled echo (GRE).^109^, ^115^, ^116^ The use of susceptibility‐weighted MRI may allow for visualization of approximately twice as many microhemorrhages and different treatment decisions compared to a GRE T2*.^117^ More data are needed to determine how sequence affects patient management in this context, with initial data suggesting that the use of susceptibility‐weighted imaging (SWI) rather than GRE may result in change in management in 4% to 5% of patients.^118^ If GRE T2* is used at one time point and susceptibility at another (or change in field strength 1.5T vs 3T), it may be impossible to tell whether a microhemorrhage is new or better seen due to differences in technique. Again, this highlights the importance of a standardized and consistent assessment over serial exams in a patient. The ASNR has published standardized guidelines for imaging protocols, interpretation, and reporting of ARIA.^30^

Cases of severe and fatal ARIA have been documented in individuals with one area of cSS on their baseline MRI. In the dose expansion part of a Brainshuttle Phase Ib/IIa study, a 78‐year‐old woman with a large occipital region of superficial siderosis on screening MRI – consistent with probable CAA diagnosis – experienced a fatal lobar macrohemorrhage.^77^ Similarly, in the TRAILBLAZER‐ALZ 2 trial, a participant with a 50‐mm area of cSS on screening MRI developed progressive ARIA after two doses of donanemab, culminating in a fatal lobar macrohemorrhage.^3^ These cases illustrate cSS on baseline MRI as a risk factor for significant CAA, and donanemab AUR recommends excluding these patients from ATT.

AI‐based algorithms have been developed for ARIA detection. Limited data suggest that these may increase reader sensitivity for ARIA, particularly for inexperienced readers and mild ARIA.^119^ In addition to identifying ARIA, these tools may aid in marking and tracking of findings (e.g., microhemorrhages) over serial exams.

## 5 ARIA CLINICAL MANIFESTATIONS AND RISK FACTORS

### 5.1 Clinical manifestations

While ARIA is a radiologic finding, it can sometimes be associated with clinical symptoms as seen in ∼3% to 6% of participants in donanemab/lecanemab Ph3 RCTs.^3^, ^4^, ^26^, ^27^ In the carefully monitored clinical trials – supported by highly experienced central readers, staff, and treating clinicians – ARIA has been primarily asymptomatic and detected incidentally on routine scheduled surveillance MRIs (∼75% of ARIA cases). A subset of ARIA events (10% to 23%) are of mild or moderate symptom severity (i.e., not incapacitating to prevent daily activities) and typically transient, usually resolving prior to or with resolution of ARIA‐E, and commonly within 3 to 4 months, or with stabilization of ARIA‐H.^3^, ^4^, ^22^, ^26^, ^27^, ^110^, ^120^, ^121^, ^122^, ^123^ However, in approximately 1% to 2% of cases, ARIA has resulted in permanent disability or death in RCTs, OLE studies, and clinical practice.^3^, ^4^, ^22^, ^26^, ^27^, ^110^, ^120^, ^121^, ^122^, ^123^ Mitigation of serious adverse events related to ARIA should be a major focus in closely monitoring patients during treatment.

**FIGURE 4:**
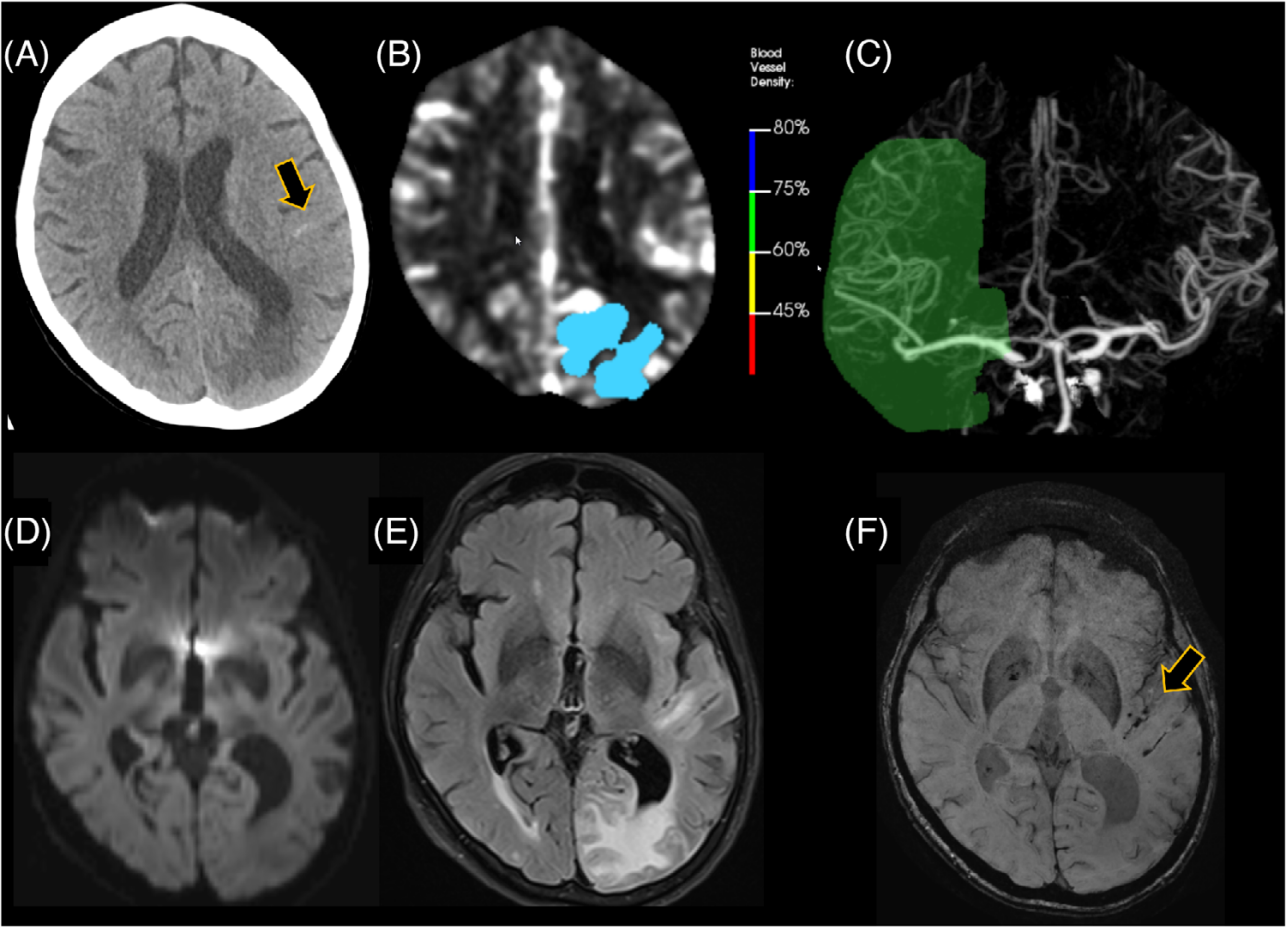
Example of amyloid‐related imaging abnormalities presenting with stroke‐like symptoms in a patient undergoing anti‐amyloid immunotherapy. A woman in her early 70s on anti‐amyloid immunotherapy presented to the ED after awakening with new aphasia, right upper extremity weakness, and confusion. (A) Non‐contrast head CT demonstrated left frontal subarachnoid hemorrhage (arrow) and left parietal vasogenic edema with cortical sparing. (B) In the left parietal lobe, hypodensity between 5 and 12 Hounsfield units (HU) was flagged (blue) by automated stroke assessment software. (C) On CTA, a 60% to 75% decrease in blood vessel density in the left middle cerebral artery distribution was flagged by software (green). (D) Subsequent MRI was negative on diffusion trace (no infarct). (E) MRI FLAIR demonstrated bilateral occipital and left temporal and parieto‐occipital vasogenic edema and sulcal effusions (ARIA‐E). (F) SWI demonstrated superficial siderosis in the left Sylvian fissure and overlying the left temporal lobe (ARIA‐H, arrow). Automated software analysis flagged abnormalities on the CT (B, C); however, these were not infarcts. MRI with diffusion (D) rules out infarct and confirms ARIA (E, F). When patients with MCI or early Alzheimer's disease present in the ED with stroke‐like symptoms, caution is needed when interpreting CT and CTA, even with automated software, which can be misleading, as in this case. ARIA‐E, ARIA manifesting as vasogenic edema; ARIA‐H, ARIA manifesting as hemorrhagic lesions; CT, computed tomography; CTA, CT angiography; ED, emergency department; FLAIR, fluid‐attenuated inversion recovery; MRI, magnetic resonance imaging; SWI, susceptibility weighted imaging; MCI, mild cognitive impairment.

Group‐level correlations exist between ARIA radiological severity and symptoms, but at the individual level, severe radiological ARIA presentations may range from asymptomatic to severe symptoms. Symptomatic ARIA typically presents with acute or subacute non‐specific symptoms such as headache, confusion, disorientation, dizziness, vertigo, nausea, vomiting, fatigue, blurred vision, visual disturbances/impairments, and gait difficulty, imbalance, or incoordination. Severe symptoms are uncommon and may include exacerbations of the above common symptoms, most often headache. Serious adverse events caused by ARIA (occurring in <1% to 2% of treated participants and <5% of ARIA events in donanemab/lecanemab Ph3 RCTs) include focal neurological deficits that may mimic stroke (Figure 4), delirium/encephalopathy, stupor, seizure, status epilepticus, and malignant hypertension.^26^, ^27^, ^110^, ^120^, ^121^, ^124^, ^125^

Presentations of ARIA may be misdiagnosed as ischemic stroke (as a clinical presentation) (Figure 4) and with subarachnoid hemorrhage or posterior reversible encephalopathy syndrome (as radiologic differential diagnosis), highlighting the importance of knowledge that a patient is receiving ATT.

There are very limited data thus far on the relationship of ARIA to long‐term cognitive or functional outcomes. One recent study with lecanemab reported that patients who had ARIA (either ARIA‐E or ARIA‐H) had a slightly lower rate of clinical worsening on the Clinical Dementia Rating Sum of Boxes (by at least three points) over 18 months in the double‐blind phase or over 36 months in the OLE phase.^126^ Additional sensitivity analyses did not find any evidence of accelerated long‐term progression for participants with ARIA versus those without ARIA.

### 5.2 Risk factors

After nearly two decades of clinical trial experience with ATTs, several risk factors for ARIA are well established, while others are emerging (Table 1). Well‐established risk factors are (1) APOE‐ε4 allelic load, (2) evidence of CAA based on the presence of microhemorrhages and cSS on baseline MRI, and (3) duration, dose, and titration time course of treatment (most ARIA occurs after initial doses, between months 2 and 6 of treatment).^26^, ^27^, ^110^, ^120^, ^121^, ^124^ Compared to APOE ε4 non‐carriers, APOE ε4 heterozygotes may have ∼1.5 to 2 times greater risk for incident ARIA‐E and similarly increased risk for ARIA‐H; APOE ε4 homozygotes may have ∼2.5 to 5 times greater risk for incident ARIA‐E and for ARIA‐H.^3^, ^4^, ^26^, ^27^, ^110^

Emerging ARIA‐E risk factors, independent of the well‐established factors (that may yet be found to interact with them), suggested by post hoc modeling analyses and from Ph3 trials and OLEs that require further study include (1) higher baseline amyloid PET burden and higher baseline cerebrospinal fluid levels of phosphorylated tau, (2) elevated baseline blood pressure (BP), (3) absence of anti‐hypertensive medication use likely reflecting untreated or suboptimally controlled hypertension, and (4) WMH severity measured by Fazekas score (Table 1).^110^, ^120^, ^121^, ^127^ Among these risk factors, elevated BP is notable as a modifiable risk factor and warrants particular attention in clinical practice. Careful assessment, monitoring, and management of BP may represent an important component of risk‐mitigation strategy in patients receiving ATT.

Other potential ARIA risk and safety‐related factors are likely to emerge with greater study of potential mechanistic interactions, for example, cross‐talk between cerebrovascular risk factors, such as BP or WMHs, CAA, and ICH, direct comparative studies between ATTs, and further data on potentially high‐impact safety risks (e.g., concomitant use of anticoagulants and ATTs and risk of ICH).^26^, ^27^, ^110^, ^120^, ^122^ For example, it may be postulated, based on reported incidence of ARIA in RCTs/OLEs or potential mechanism of action (e.g., greater impact on soluble than insoluble amyloid species and plaques), that ARIA risk may be lower for some ATTs than others.^122^ However, differing RCT/OLE inclusion/exclusion criteria and treatment paradigms prohibit direct comparisons of ARIA between plaque‐lowering ATTs in the absence of well‐designed and executed comparative studies.^27^, ^110^

## 6 ARIA IMPLICATIONS FOR REAL‐WORLD CLINICAL PRACTICE

### 6.1 Patient selection and interdisciplinary care and system requirements

Optimal mitigation of ARIA, as part of safe, effective, and gradually increasing adoption and implementation of ATTs in real‐world clinical practice, requires appropriate shared decision‐making and patient selection; knowledgeable and coordinated interdisciplinary care and management (including among prescribing specialists/subspecialists, primary care, neuroradiology, neuropsychology, emergency medicine, hospitalists, neurology/vascular neurology, intensivists, infusion centers); rigorous processes and protocols; and substantial commitment and investment by healthcare practices and systems.^26^, ^27^, ^28^, ^29^, ^128^ A large majority of practicing clinicians, including cognitive‐behavioral/dementia subspecialists, do not yet have first‐hand experience detecting, diagnosing, or managing ARIA. In addition to knowledge and proficiency gaps, systemic readiness challenges remain due to interrelated complexities and constraints in access, coverage, costs, logistics, and resources in real‐world settings outside highly specialized tertiary/quaternary AD/dementia research and care centers.

Guidance and considerations regarding clinical use of ATTs and ARIA can be found in FDA‐PI/labels, ADRD Therapeutic Work Group AURs, American Academy of Neurology (AAN) guidance, American Heart Association (AHA) vascular neurology considerations, and ASNR ARIA imaging recommendations and practice considerations.^26^, ^27^, ^28^, ^29^, ^30^, ^31^, ^113^, ^114^, ^120^, ^128^ The ATT FDA‐PI includes a Box Warning for ARIA that cautions regarding higher and more impactful risk in ε4 homozygotes and that risks should be discussed, and testing for APOE ε4 status should be performed prior to ATT initiation. Overall, the ATT FDA‐PI is also less specific, proscriptive, and restrictive compared to AURs and other guidance regarding patient selection and inclusion/exclusion criteria and ARIA detection, monitoring, and management protocols.^26^, ^27^, ^28^, ^29^, ^30^, ^31^, ^110^, ^113^, ^114^, ^120^, ^128^

In Europe, EMA European public assessment reports (EPARs) and summary of product characteristics (SmPCs) highlight ARIA as a very common risk. As noted earlier, ATT use is restricted to individuals with 0 to 1 APOE ε4 alleles, and post‐authorization safety studies are mandated. In the UK, the MHRA guidance also requires APOE genotyping prior to treatment initiation, lists baseline MRI‐diagnosed CAA as a contraindication, and requires controlled‐access registration, post‐authorization safety studies, and dissemination of risk‐minimization materials.

In the United States, a recent comparison of lecanemab implementation across two distinct healthcare settings – an East Coast private neurology practice and a West Coast academic memory clinic involving a total of 165 patients – reported average ARIA‐E and ARIA‐H rates of 8% and 7%, respectively, which is lower than those observed in the CLARITY‐AD trial.^129^ Over the course of 1 year of treatment, no deaths were reported, although three cases of symptomatic ARIA required urgent medical intervention (two from the academic memory clinic and one from the private neurology practices).^129^ Additional real‐world data from a multi‐site case series of 178 lecanemab‐treated patients across nine US sites (mean of 25 doses; about 1 year of treatment) reported 23 ARIA events. Most events were radiographically mild, while two cases were clinically symptomatic, presenting with headaches. No microhemorrhages, ICH, or deaths were reported.^130^ While these data suggest that ATT implementation may be achievable with manageable risk in some structured clinical environments, they must be interpreted cautiously, given the variability across practice settings. The workgroup is aware of and is continuously monitoring emerging safety reports from post‐marketing experiences and fatal cases reported in the FDA Adverse Event Reporting System (FAERS) database. Outside highly controlled clinical trial environments, continued vigilance and systematic post‐authorization surveillance remain essential.

### 6.2 ARIA management

**FIGURE 5:**
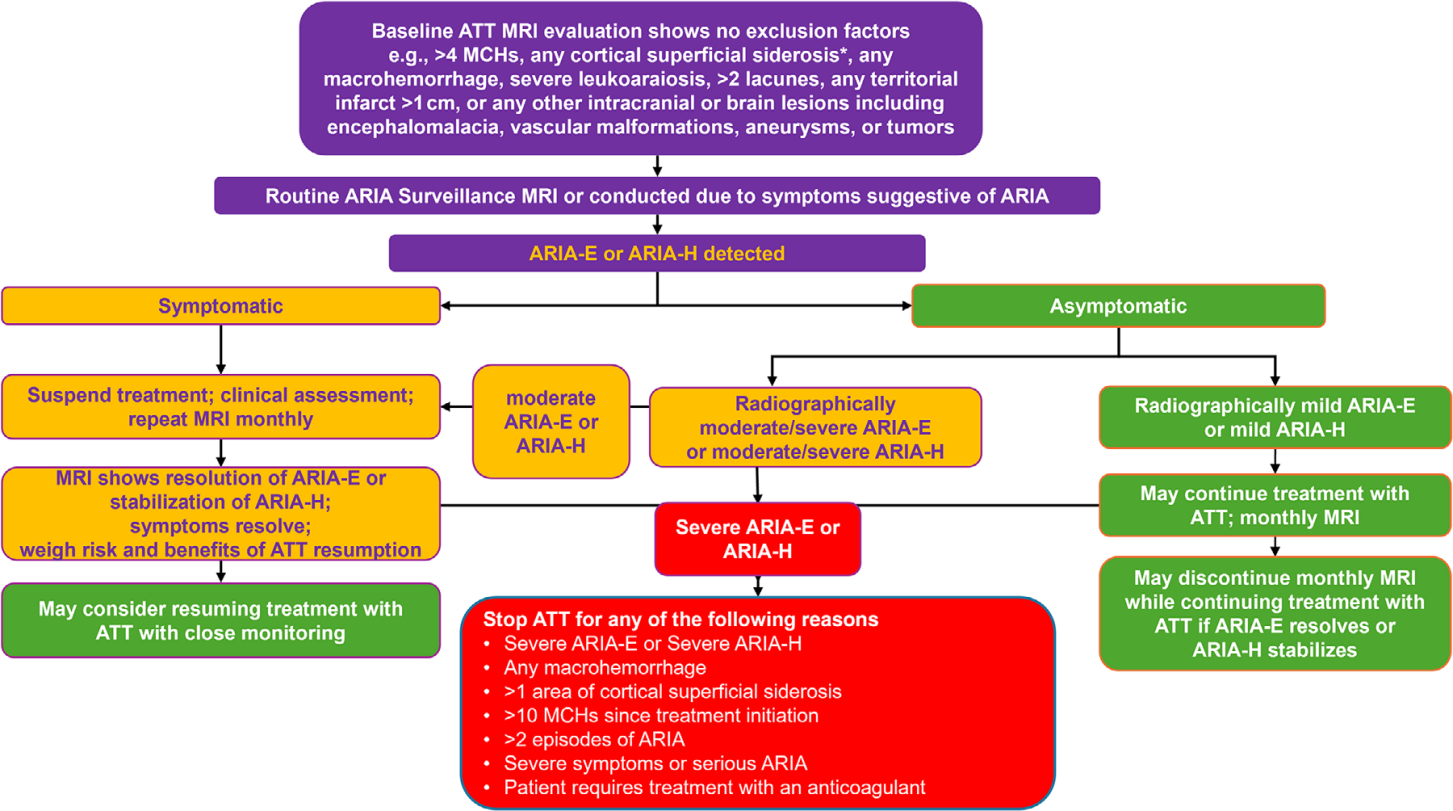
Schematic flow chart for management of amyloid‐related imaging abnormalities. Adapted from lecanemab AUR, Cummings et al., and donanemab AUR, Rabinovici et al.^26^, ^27^ *Note that the donanemab AUR includes any cSS as an exclusion criterion, while the donanemab Phase 3 trial (TRAILBLAZER‐ALZ 2) allowed one cSS. ATT, amyloid targeting therapies; AUR, appropriate use recommendation; cSS, cortical superficial siderosis; MCH, microhemorrhage.

Existing recommendations provide baseline and ARIA/ATT surveillance/monitoring MRIs, reporting and workflows (including guidance and considerations regarding standardization of field strength, sequences, consistency, reporting templates, availability of prior images, and timing of scans), and management schema.^26^, ^27^, ^28^, ^29^, ^30^, ^31^ Figure 5 provides a schematic for ARIA management based on the presence or absence of symptoms and radiographic severity. However, clinical judgment is always paramount as no set of recommendations can fully anticipate or reproduce the complexities of clinical practice; thus, clinicians should always consider individual‐level factors when prescribing ATTs and managing ARIA (e.g., ARIA nature, severity, symptoms, history, evolution, comorbidities, concurrent medications, and APOE genotype).^26^, ^27^ Additionally, management recommendations for severe or serious ARIA‐E include prompt evaluation, brain MRI, consideration of timely high‐dose glucocorticoid treatment (e.g., methylprednisolone 1 g/day IV for 5 days followed by an oral steroid taper over several weeks) based on data from CAA‐ri and close monitoring and precautions, including vital signs, neurological status, seizures, and other potential complications.^22^, ^26^, ^27^, ^28^, ^29^, ^30^, ^31^, ^110^, ^120^, ^127^, ^128^, ^131^, ^132^

### 6.3 ARIA and concomitant antiplatelet therapy, anticoagulation, and thrombolytics

While concomitant antiplatelet monotherapy at standard dosages (e.g., aspirin up to 325 mg daily) has not emerged as an ARIA risk factor, safety data regarding anticoagulation remain limited, and the use of thrombolytics in persons on ATTs appears to carry extreme risk. Several deaths of persons on ATTs following anticoagulation or administration of thrombolytics have been reported.^3^, ^24^, ^27^, ^110^, ^120^, ^123^ Anticoagulation likely increases the risk of severe ARIA‐H in the form of symptomatic ICH, a condition associated with very high morbidity and mortality risk.^22^, ^26^, ^27^, ^28^, ^29^, ^120^ ATT FDA‐PI urges caution regarding concomitant anticoagulation, while AURs list anticoagulation as exclusionary for initiating or continuing ATTs until additional safety data are available.^26^, ^27^, ^28^, ^29^, ^113^, ^114^ In Europe and the UK, anticoagulation use is listed as a contraindication.

Focal neurologic deficits due to ARIA‐E may clinically mimic acute ischemic stroke (Figure 4). At least three cases of ARIA‐E presenting with acute neurological deficits mimicking ischemic stroke and subsequently receiving thrombolysis have resulted in fatal hemorrhagic outcomes. A 72‐year‐old APOE4 heterozygous participant in the long‐term extension of TRAILBLAZER‐ALZ 2, who was treated with donanemab, presented with headache and slurred speech 7 days after a fifth dose and developed bilateral ICH following IV tenecteplase. Pre‐thrombolysis CT and perfusion imaging were unremarkable, whereas post‐treatment MRI revealed severe ARIA‐E, superficial siderosis, and multifocal lobar hemorrhages.^133^ In the modified titration study of TRAILBLAZER‐ALZ 6 (NCT05738486), an APOE ε4 heterozygous patient experienced fatal hemorrhage after receiving tenecteplase for presumed stroke, where initial hypodensity on CT was retrospectively recognized as ARIA‐E.^123^ A similar fatal event occurred in an APOE ε4 homozygous patient in the OLE of the lecanemab CLARITY trial following alteplase administration,^24^ with neuropathology examination revealing multifocal ICH and severe CAA‐ri (Section 3.1.2). Consistent with these observations, the donanemab FDA‐PI Box Warning says that treating clinicians should consider whether focal neurological stroke‐like symptoms could be due to ARIA‐E before potentially administering thrombolytic therapy. Pending data to demonstrate safety, AHA guidance states, “treating clinicians might reasonably consider a high level of caution or avoidance of thrombolytic use in patients receiving” ATTs, and AURs recommend that persons on ATTs not receive thrombolytics.^26^, ^27^, ^110^, ^113^, ^120^ Mechanical thrombectomy without thrombolytics does not appear to increase ICH risk and should be considered for patients experiencing acute large vessel occlusive stroke.^27^, ^110^, ^120^

The AURs state that mitigating potentially life‐threatening complications of severe or serious ARIA is a rationale for more conservative recommendations that prioritize patient safety in the still‐early roll‐out of ATTs into real‐world practice, where implementation gaps exist, and provide details on considerations for clinical resources, workflows, standard operating procedures (SOPs), and electronic health record (EHR) templates. Similar considerations, guidance, and details are discussed in the AAN, AHA, and ASNR guidance documents, and by others.^26^, ^27^, ^28^, ^29^, ^30^, ^31^, ^110^, ^120^, ^128^ AURs and association/specialty association guidance documents acknowledge that recommendations and considerations will evolve as data from trials and real‐world practices accrue, including from patient registries such as the Alzheimer's National Registry for Treatment and Diagnostics (ALZ‐NET) (www.alznetproviders.org), UK/EU regulatory data collections, and the International Registry for Alzheimer's Disease and Other Dementias (InRAD) initiative.

## 7 RISK/BENEFIT CONVERSATIONS IN TREATMENT DECISION‐MAKING AND PATIENT PERSPECTIVE

The approval of ATTs and the consistently observed elevated risk for ARIA among APOE ε4 carriers necessitated a shift in practice guidelines for individuals with MCI and dementia. Expert commentaries^134^ and AURs^26^, ^27^ now support the performance of APOE genetic testing as a component of the work‐up when considering ATTs. APOE testing was not previously recommended, but this information is now essential for thorough informed consent of individuals being considered for treatment – though testing and disclosure should be recommended but not required. Clinicians will need to be prepared to engage in a multistep^135^ process of evaluating appropriateness for treatment, with each step involving education and counseling. Particularly since this involves genetic testing and carries potential implications for family members,^134^ providers may vary in their comfort with these elements and may benefit from involving genetic counselors.

As noted, the FDA‐PI Box Warnings for lecanemab and donanemab emphasize increased ARIA risk among APOE ε4 homozygotes; but the labels do not exclude these patients from treatment. As noted earlier in Section 1, some national regulatory agencies, such as the UK's, have approved ATTs only for individuals who carry one or no copies of APOE ε4 allele.^12^, ^136^ Similarly, the US Veteran's Affairs hospital system refused to approve treatment for APOE ε4 homozygotes.^16^ Presumably, these decisions are based on a perceived differential risk/benefit ratio that includes not only the increased risk for ARIA but preliminary indications that treatment benefit may be reduced or even null in this genetic subgroup.^3^, ^4^ Whether the observed reduced efficacy is due to genetic background versus some other factors, such as baseline amyloid or tau burden, or lower exposure due to ARIA‐related treatment interruptions, however, remains unknown. Moreover, the total sample sizes for these subgroups within trials were small, lowering precision and emphasizing the need to pursue meta‐analytic approaches to interrogate the role of various risk elements, for safety and efficacy outcomes.

Patients and their families value the opportunity to maintain cognitive performance, functional independence, quality of life, and autonomy.^137^, ^138^ For some, the potential to slow disease progression and delay functional decline will justify even the estimated increased risk of ARIA for APOE ε4 homozygotes. Educating and counseling ε4 homozygotic patients and their families about treatment risks and benefits may be particularly challenging, but the workgroup recommends caution in decisions about absolute restrictions from treatment until such time as a more thorough understanding of the pathophysiological mechanisms of ARIA and the relative risks and benefits of treatments can be elucidated.

## 8 RECOMMENDATIONS AND FUTURE DIRECTIONS

Building on the 2011 AARR workgroup recommendations, this work reflects decades of experience with AD clinical trials and the transition of ATT into clinical practice. Drawing upon subject‐matter expert deliberations and a synthesis of peer‐reviewed literature and publicly available data, we provide an updated framework of ARIA understanding, with particular attention to real‐world implementation.

### 8.1 Recommendations

A summary of workgroup recommendations regarding ARIA terminology, mitigation of serious adverse events, treatment indications, and antithrombotic agents and stroke treatment considerations is provided in Box 1.

**Box 1: Recommendations from the workgroup**

**Terminology**

- ARIA terminology should specifically refer to MRI signal abnormalities that arise in the context of ATTs, with the aim of facilitating timely and targeted communication of findings to referring physicians in treated individuals.
- Use of the term “spontaneous ARIA” is discouraged. Radiologic findings resembling ARIA in placebo groups or untreated individuals should be described and interpreted (e.g., microhemorrhage, superficial siderosis) according to the specific radiologic features observed.
- If a patient's treatment status (e.g., ongoing participation in a blinded clinical trial) is unknown and imaging shows evidence consistent with edema or effusion, then ARIA‐E should be considered and described in accordance with ASNR and AUR recommendations.^26^, ^27^, ^28^, ^29^, ^30^, ^31^

**Mitigation of serious adverse events related to ARIA**

- Prioritize safety in ATT therapy. The potential for serious or life‐threatening ARIA, particularly during early real‐world implementation when practice gaps remain, supports more conservative recommendations centered on patient safety within an informed, person‐centered, shared decision‐making framework.
- Only practitioners with sufficient clinical specialty background and proficiency and resources to initiate and manage ATTs should prescribe and manage ATTs.
- Apply evidence‐ and AUR‐informed individualized risk‐benefit assessment, person‐centered, shared decision‐making and partnerships, and rigorous monitoring strategies; these are especially critical elements for individuals with a higher risk profile (see Table 1 for ARIA risk factors).
- Careful coordination and close communication with emergency personnel and acute‐care teams (e.g., emergency medicine, radiology, neurology) are needed to ensure awareness of ATT exposure in the setting of potential stroke and avoidance of inappropriate use of thrombolysis if ARIA is in the differential.
- With appropriate patient selection, monitoring, and management, most ARIA is radiographically mild or moderate in severity; there should be a focus on mitigation of preventable serious ARIAs.

**Treatment eligibility and CAA**

- ATT has not been shown to improve the course of CAA and is associated with increased risk of adverse effects in this setting. ATT should therefore not be used to treat CAA outside a research setting.
- In line with AUR,^26^, ^27^, ^28^, ^29^ patients with early‐stage clinical (or symptomatic) AD otherwise eligible for passive ATT should be excluded from treatment in clinical practice if they have prior ICH or convexity subarachnoid hemorrhage, more than four cerebral microbleeds, or cortical superficial siderosis.

**Antithrombotic agents and stroke treatment**

- Anticoagulation may increase the risk of severe ARIA‐H, especially in the form of symptomatic ICH; in the absence of definitive evidence that anticoagulation does not increase the risk of serious ARIA‐H, concomitant use with ATT should be avoided.
- In suspected stroke in persons receiving ATTs where thrombolytic use is under consideration, rapid emergency brain MRI should be performed whenever feasible to exclude ongoing ARIA. ARIA‐E‐related focal neurologic symptoms may mimic ischemic stroke and should be considered in the differential diagnosis before thrombolysis (e.g., tissue plasminogen activator or tenecteplase), as administration of thrombolytics in individuals receiving ATT appears to carry a very high risk of disability or fatal outcomes (several fatal cases following thrombolytic administration in this setting have been reported). Thrombolytics should generally be avoided in persons receiving ATT.
- In cases of acute ischemic stroke caused by large vessel occlusion in a person receiving ATT, mechanical thrombectomy without thrombolytic therapy may not increase the risk of ICH and should be considered.

These recommendations are intended to inform the field broadly but do not represent a clinical practice guideline or prescriptive care pathway.

### 8.2 Outstanding challenges and future directions

As ATTs become increasingly utilized in clinical practice, addressing the outstanding challenges related to ARIA is critical for optimizing patient care and advancing our understanding of these imaging phenomena. Several key areas require further exploration and refinement to improve the detection, interpretation, and management of ARIA. Priority research areas identified by the workgroup are summarized in Box 2.

**Box 2: Proposed research priorities**

**Mechanistic understanding of ARIA and preclinical models**

- Develop and refine animal models that better recapitulate ARIA mechanisms.
- Expand neuropathologic and *post mortem* studies to improve mechanistic understanding of ARIA.
- Enhance imaging and biomarker characterization of CAA and leverage CAA‐ri as a translational model to better characterize the underlying mechanisms of symptomatic ARIA.
- In addition to APOE ε4 genotype, clarify the role of APOE ε2 in ARIA.
- The relationship of ARIA to brain volume reduction requires further study.
- Clarify immune system contribution and complementary pathways to ARIA development.
- Understand the mechanisms underlying ARIA rates and safety profiles of alternative routes of ATT administration and CNS entry, such as targeting of the transferrin receptor and subcutaneous formulations.

**ARIA detection**

- Develop and validate techniques for early and more accurate detection of mild radiographic or evolving ARIA.
- Broaden implementation of standardized neuroimaging approaches and protocols.
- Clarify the role of SWI versus GRE sequences, field strength, and platforms for CAA and ARIA‐H detection, monitoring, and management.
- Investigate the role of blood‐based and other biomarkers in ARIA to aid in risk stratification, detection, monitoring, and management.
- Advance automated detection algorithms and expand training resources for neuroradiologists.

**Treatment**

- Focus on the prevention of serious and symptomatic ARIA and continue to evaluate the association of ARIA with long‐term clinical outcomes.
- Assess existing and generate new safety data on the concomitant use of antithrombotic agents.
- Continue to reevaluate eligibility criteria for therapy initiation, dosing, and redosing (e.g., thresholds for cerebral microhemorrhage counts), considering microhemorrhage timing, phenotype, and anatomic distribution.
- Evaluate new biomarker‐informed paradigms and regimens (e.g., dose, dose titration, flexible dosing with step up/down) for ATT initiation, management, and dose suspension and discontinuation, especially for high‐risk subgroups, groups not currently broadly included in practice (e.g., >4 microhemorrhage or >1 or 2 superficial siderosis, on anticoagulation, high leukoaraiosis burden, or evidence of substantial cerebrovascular disease), or groups currently discontinued from treatment due to accumulating ARIA and risks (e.g., >9 incident microhemorrhage or >2 focal areas of superficial siderosis, recurrent ARIA‐E with >2 episodes of ARIA‐E, started on anticoagulation during ATT therapy), with very close safety monitoring.
- Evaluate ATT impact on groups with AD and co‐pathology (e.g., Lewy body disease, vascular contributions to cognitive impairment and dementia).
- Assess optimal management strategies for suspected stroke in the context of ATT.
- Evaluate and develop evidence base to refine treatment approaches (e.g., timing, dosing, and duration of corticosteroid or other regimens) for severe/symptomatic ARIA or those with ARIA and potentially high risk of developing serious ARIA (e.g., high‐risk persons with repeated or expanding ARIA in the same regions).
- Assess ARIA risk and monitoring in persons with treatment‐related amyloid clearance (TRAC) who are maintained on long‐term ATT.

**Generalizability**

- Increase representation of understudied and highest risk populations in clinical trials and real‐world data collection, such as individuals with diverse socioeconomic backgrounds and those with multiple comorbidities.
- Address patient‐level access barrier, such as limited availability of ARIA screening and monitoring tools outside academic centers.

**Data sharing/real‐world evidence**

- Encourage reporting of all serious ARIA cases, ideally through centralized data registries (such as ALZ‐NET or similar registries).
- Strengthen interdisciplinary collaboration among researchers, clinicians, industry, and regulatory stakeholders to expand real‐world data collection and evidence generation; assess pooled data from completed clinical trials.

A significant challenge in ARIA research is the standardization of imaging techniques across clinical trials and real‐world settings. The variability in MRI scanner technology and imaging sequences used for ARIA detection in general clinical practice means that serial safety scanning within a given person should be performed on a similar MRI platform, which can be logistically challenging. The relative roles of SWI versus GRE for the detection of microhemorrhages warrant further evaluation. Moreover, the increased demand for imaging interpretation in the setting of ATT implementation poses challenges for radiology services, highlighting practical solutions such as automated detection algorithms and enhanced training for neuroradiologists. From a patient perspective, accessibility for those who do not live near an academic center can complicate routine screening for ARIA.

Despite improvements in imaging detection, *post mortem* validation of ARIA findings remains an unmet need. Access to brain tissue from ARIA cases is limited, and factors such as time since the last treatment dose must be carefully considered in *post mortem* analyses. Better animal models are also needed to study ARIA pathophysiology and explore potential interventions, as current models incompletely recapitulate human pathology.

Another challenge is the lack of imaging techniques that can directly visualize CAA in vivo. Currently, amyloid‐PET does not reliably distinguish parenchymal amyloid from CAA at the individual‐patient level, although this is an active research field.^139^ On MRI, CAA‐related hemorrhagic markers are downstream sequelae rather than direct visualization of vessel‐wall Aβ, so small/early lesions may be missed or misclassified.^39^, ^140^ Developing such techniques would enhance our ability to study ARIA in the context of underlying vascular amyloid pathology.

Novel strategies like Brainshuttle technology offer a potentially promising approach to mitigating ARIA.^103^ By improving drug delivery across the BBB via transferrin receptor‐mediated transcytosis, this method may reduce vascular amyloid interactions and enhance therapeutic efficiency, potentially lowering the risk of ARIA in future ATTs. Other strategies, such as modified titration scheduling of ATT and alternative routes of administration (e.g., SC), may further contribute to ARIA risk mitigation while preserving therapeutic efficacy.

Treatment eligibility criteria, particularly regarding the acceptable number of lobar microhemorrhages, may also require re‐evaluation. Furthermore, additional safety data are needed, particularly for patients with more than four microhemorrhages, as observed in some OLE trials.

A clearer understanding of ARIA risk – including data on asymptomatic cases – is critical for more comprehensive risk‐benefit assessments. Precision risk prediction should focus on absolute risk for specific outcomes (e.g., asymptomatic vs symptomatic ARIA) using combinations of key factors such as APOE genotype, microhemorrhage burden, and white matter disease severity.

Data from prevention and pre‐symptomatic trials suggest that ARIA‐E can occur even at the earliest biological stages of AD, but also seems to be antibody‐, dose‐, and APOE genotype‐specific. In DIAN‐TU, solanezumab exposure was not associated with ARIA, whereas gantenerumab showed dose‐dependent ARIA‐E risk – with most events mild, reversible on MRI, and often asymptomatic. These results suggest that ARIA can occur before clinical symptoms emerge and that, in this group, risk stratification will also be necessary.^141^

Recent multivariate analyses from the donanemab trials have identified independent ARIA‐E risk factors, including more than two microhemorrhages, cSS, elevated amyloid PET burden, and high mean arterial pressure (MAP), beyond the contribution of APOE ε4 status.^121^ Encouragingly, studies with donanemab have suggested that anti‐hypertensive treatment^121^ and a more gradual up‐titration of the dose may reduce ARIA‐E risk.^123^ Similarly, GRADUATE trial data showed that white matter abnormalities (as measured by Fazekas score) independently contribute to ARIA risk.^127^ Importantly, these analyses found no evidence of long‐term cognitive or functional decline in most participants who experienced ARIA‐E. Effects appear transient and manageable with protocolized monitoring and dose adjustments.^127^ Continued research is needed to refine these risk models, especially for predicting symptomatic or rare serious events, which will likely require pooled data from multiple programs.

There is also a critical gap in the data from individuals with multiple comorbidities or those from diverse socioeconomic backgrounds, limiting the generalizability of current findings.

Long‐term clinical outcomes associated with ARIA remain to be elucidated. While there is concern over possible adverse clinical effects, emerging evidence suggests that ARIA‐E, particularly when asymptomatic, may be associated with more effective amyloid clearance and potentially better clinical outcomes.^126^ Reports from gantenerumab and lecanemab trials indicate that non‐serious ARIA cases may have similar – or even superior – clinical responses to therapy.^127^, ^130^

Beyond risk assessment, management of ARIA requires greater clinician awareness and the use of standardized management protocols. Key research priorities regarding treatment are outlined in Box 2 and include strengthening safety data on concomitant antithrombotic use; refining eligibility and dosing criteria for ATT initiation, dosing, and redosing (including evaluation of new biomarker‐informed paradigms especially in high‐risk subgroups); improving management of suspected stroke in the context of ARIA; evaluating treatment options on groups with co‐pathologies; advancing approaches to prevent serious ARIA while clarifying its long‐term clinical impact; refining treatment approaches (e.g., regarding corticosteroids) for severe/symptomatic ARIA or those with ARIA and potentially high risk of developing serious ARIA; and assessing ARIA risk and monitoring in persons with TRAC who are maintained on long‐term ATT.

Finally, progress in these areas will depend on interdisciplinary collaboration among researchers, clinicians, academicians, regulators, and industry representatives to refine our understanding of ARIA and improve patient outcomes. Real‐world registries, such as ALZ‐NET and InRAD, will play a pivotal role in this process by providing longitudinal, real‐world evidence on ATT use and associated ARIA events.

## AUTHOR CONTRIBUTIONS

All authors contributed to the writing of the manuscript and critically revised and approved the final version. Alireza Atri drafted Table 1 and Figure 5. Tammie L.S. Benzinger and Petrice M. Cogswell drafted Figures 1 and 4. James A. R. Nicoll drafted Figure 2. Cynthia A. Lemere drafted Figure 3.

## CONFLICT OF INTEREST STATEMENT

E. van Etten reports a research contract with Alnylam Pharmaceuticals Inc. to recruit patients for a clinical trial paid to Leiden University Medical Center. She also serves as an unpaid member of the steering committee for the clinical trial of Alnylam Pharmaceuticals Inc. and served as an advisor/consultant to Biogen, paid to Leiden University Medical Center.

Simin Mahinrad is a full‐time employee of the Alzheimer's Association. Travel related to her attendance at meetings or conferences is covered by her employer.

Joshua D. Grill received grant funding from the National Institute of Aging, the Alzheimer's Association, the BrightFocus Foundation, Eli Lilly, Genentech, and Eisai. In addition, he has received consulting fees from SiteRx, and support for attending meetings and/or travel from the Alzheimer's Association.

Stephen Salloway reports research support for conducting clinical trials from Lilly, Biogen, Genentech, Avid, Roche, Eisai, and Novartis. He has received consulting fees from Lilly, Biogen, Roche, Genentech, Jansen, Acumen, BMS, Novo Nordisk, AbbVie, and Neurophet. He is an Associate Editor of the *Journal of Prevention of Alzheimer's Disease* and *Alzheimer's and Dementia: Diagnosis, Assessment and Disease Monitoring* since 2014.

Alireza Atri serves on several Alzheimer's Association workgroups on a pro bono basis and has not received compensation or honoraria for his participation in any workgroup, including his involvement in this ARIA Workgroup. He has received meeting support from the Alzheimer's Association for unrelated meetings/conferences. He received grant funding from Alzheon, Athira, Biogen, Biohaven (with ADCS), Eisai (with ATRI/ACTC), Lilly (with ATRI/ACTC), Vivoryon (with ADCS), ACTC, ADCS, AZ Alzheimer's Research Consortium and AZ DHS, ATRI, GAP, the University of Southern California (USC), Indiana University, Johns Hopkins University, Washington University St. Louis, Gates Ventures, AZ DHS, National Institute on Aging (NIA)/National Institutes of Health (NIH), and FNIH. He holds royalties or licenses for a book on dementia published by Oxford University Press. He has received consulting fees from Lundbeck, Novo Nordisk, Eisai, Prothena, Roche/Genetech, Merck, ONO, AriBio, Vaxxinity, Life Molecular Imaging, and Axome. He has received payment or honoraria for lectures, presentations, or educational events from Eisai and Lundbeck. He has received travel or meeting support specifically for consulting meetings, workgroup sessions, scientific/medical presentations, or educational programs from the Alzheimer's Association, Alzheimer's Disease International (ADI), and the AAN. He has participated on a Data Safety Monitoring Board or Advisory Board for Roche/Genetech.

Petrice M. Cogswell has received consulting fees from Eli Lilly & Co., payment for medical education presentation from Eisai Inc., and payment for CME activity from Kaplan, Medical Learning Institute, and PeerView. She has received travel payment from the American Academy of Neurology for scientific presentations. She has also served as co‐chair of American Society for Neuroradiolgy (ASNR) Alzheimer's, ARIA, and Dementia study group (no compensation). In addition, she serves on the Data Safety Monitoring Board of Eisai and Eli Lilly, where no payments will be made to her or her institution.

Tammie L.S. Benzinger reports the following: grants or contracts to Washington University; grants or contracts were received from the National Institutes of Health (NIH), the Alzheimer's Association, and the ASNR: from Siemens with funds paid to her institution; technology transfer and precursors for radiopharmaceuticals from Avid Radiopharmaceuticals/Eli Lilly, LMI, and Lantheus; scanner loan from Hyperfine to her institution. Personal compensation: Dr. Benzinger has received consulting fees from Biogen, Eli Lilly, Eisai, Bristol Myers Squibb, Johnson&Johnson, and Merck. Dr. Benzinger has received payment for CME activity from Radiology Today, Medscape, PeerView, and Neurology Today. Travel: Dr. Benzinger has received travel support from Janssen, Eisai, Cedars Sinai Medical Center, Hong Kong Neurological Association, the Radiological Society for North America, the American College of Radiology and the Alzheimer's Association. Unpaid activities: Dr. Benzinger has participated in an advisory board for Siemens (no payments). She has served as an external advisor for NIH‐funded studies (no payments). She has served as the co‐chair of ASNR Alzheimer's, ARIA and Dementia Study Group, and RSNA Quantitative Imaging Committee (QuIC) (all unpaid). She has served as a committee member of the American College of Radiology/ALZ NET imaging, NIH CNN Study Section Chair, and had a leadership or fiduciary role in the ACR Commission on Neurology (all unpaid). Patents: Dr. Benzinger reports the following patents planned, issued, or pending: US patent 16/097, 457 (DIFFUSION BASIS SPECTRUM IMAGING (DBSI), A Novel Diffusion MRI Method Used to Quantify Neuroinflammation and Predict Alzheimer's Disease (AD) Progression), and US Patent 12,016,701 (Quantitative Differentiation of Tumor Heterogeneity Using Diffusion MR Imaging Data). For additional references regarding her disclosures, see Sunshine ACT reporting: https://openpaymentsdata.cms.gov/physician/850680.

Takeshi Iwatsubo received honoraria for lectures from Eli Lilly and Eisai.

Costantino Iadecola received the following grants from NIH, all paid to his institution: RF1‐NS128947, R01‐NS095441, 1R01‐NS126467, and R01‐NS/HL37853. In addition, he has received payment for participation on the advisory board of Broadview Ventures.

Cynthia A. Lemere has received grants from the NIH (NIA, NINDS), NASA, Cure Alzheimer's Fund, and Apellis Pharmaceuticals (sponsored research), all paid to her institution. She has received consulting fees from Acumen Pharmaceuticals, ADvantage Therapeutics, Alnylam Pharmaceuticals, Apellis Pharmaceuticals, Biohaven Pharmaceuticals, Cyclo Therapeutics, Eli Lilly & Co, Merck, MindImmune Therapeutics, Novo Nordisk, Receotive Bio, and Switch Therapeutics. She has received honoraria for invited seminars from AC Immune, Alnylam Pharmaceuticals, Boston University School of Medicine, Cyclo Therapeutics, Merck, Michigan State University, Novo Nordisk, Sanofi, and UCI. She has received Honoraria for grant proposal reviews from NIH, BrightFocus Foundation, Brookhaven National Laboratory, and Cure Alzheimer's Fund. She received an honorarium from the Kenes Group for serving as an AD/PD Meeting Executive Organizer. She has received travel support to attend meetings from the Alzheimer's Association, Biohaven Pharmaceuticals, the BrightFocus Foundation, Kenes Group (AD/PD Meeting), Luxembourg National Research Fund, Merck, Novo Nordisk, and Sanofi. She has served on the Scientific Advisory Board of the Alzheimer's Association US POINTER and is a member/advisor of the DIAN‐TU Therapeutic Evaluation Committee, DSMB of LuMIND, and the External Advisory Boards of NIH MARMO‐AD and MODEL‐AD. She has served as a member, chair, past chair, and alumna of the Alzheimer's Association Medical and Scientific Advisory Group (MSAG) and is a co‐organizer of Alzheimer's FastTrack Workshop for BrightFocus Foundation and a member/advisor for Cure Alzheimer's Fund Research Leadership Council. She has a small number (less than 1%) of unexercised stock options from Acumen and MindImmune, neither of which has a commercialized product. Her husband owns Gerald Cox Rare Care Consulting, LLC.

James A.R. Nicoll received support (travel, accommodation, and subsistence) from the American Heart Association to present at the International Stroke Conference in Phoenix, Arizona, 2024.

Steven M. Greenberg received research grants from the NIH and author royalties from UpToDate. In addition, he has participated in the Global Steering Committee of Alnylam, where payments were made to his institution, and participated in the Safety Monitoring Committee of Washington University, Bayer, and Bristol Myers Squibb.

Maria C. Carrillo is a full‐time employee of the Alzheimer's Association and has a daughter in the neuroscience program at USC. She reports receiving grants from NIA and CDC. As a full‐time employee of the Alzheimer's Association, all her travel for attending meetings and/or conferences is covered by her employer. She participated in a Data Safety Monitoring Board or Advisory Board of NIA‐ and NINDS‐funded initiatives, including ADSP; served on the board of GHR Foundation, and a Research Committee at the American Heart Association (unpaid).

Clifford R. Jack Jr received grant funding from the National Institute of Health. He has also served on a DSMB for Roche on a pro bono basis, for which no payments were made to him or his institution (Mayo Clinic).

Reisa A. Sperling has received grant funding from the Alzheimer's Association, NIH, and the GHR foundation, all paid to her institution. She has also received research funding to clinical trial sites from Eli Lilly and Eisai. She has received consulting fees from AbbVie, AC Immune, Acumen, Alector, Apellis, Biohaven, Bristol Myers Squibb, Genentech, Ionis, Janssen, Nervgen, Oligomerix, Prothena, Roche, Vigil Neuroscience, and Vaxxinity. Her spouse has received consulting fees from Novartis, Merck, Janssen, and Sanofi. She has received travel reimbursement from the Alzheimer's Association, Clinical Trials in Alzheimer's Disease, and Janssen. Author disclosures are available in the Supporting Information.

## Supporting information



Supporting Information
